# Supplementary material for: Exploring effector protein dynamics and natural fungicidal potential in rice blast pathogen Magnaporthe oryzae
Source: PLoS One. 2025 Jan 24;20(1):e0307352. doi: 10.1371/journal.pone.0307352 (PMC11761166; doi:10.1371/journal.pone.0307352)
Supplement: S1 Table — (DOCX) [file pone.0307352.s001.docx]

**Supplementary table S1:** Anti-viral and insecticidal Activity plant metabolites

| SL No. | Metabolites | Class | PubChem CID | Source | Biological Effects | References |
| --- | --- | --- | --- | --- | --- | --- |
|  | Rotenone | Isoflavonoid | 6758 | *Derris elliptica* | Insecticide, anti-bacterials, anti-virals, anti-malarials, and anti-inflammatories, anti-fungal, herbicide, piscicide etc. | (Castro et al., 2010) |
|  | Ajoene | Organosulfur | 5386591 | *Allium sativum*(Garlic) | Act as anti- viral agent ( inhinit viral replication during early stage) virucidal activity against herpesviruses, anti- cancer,anti- fungal,anti- bacterial,anti- parasitics and strong insecticide | (Thomaz et al., 2008) |
|  | Allicin | Organosulfur compounds | 65036 | *Allium sativum* | Lower cholesterol and blood pressure, prevent blood clots, and improve blood circulation,anti-cancer, chemopreventive and anti-microbial properties | (Li et al., 2022) |
|  | Berberine | Alkaloids | 2353 | *Berberis aristate (*Tree turmeric) | Act as anti-viral agent,more effective against fungi,(inhibits viral DNA, inhibit cell proliferation)anti-diarrheal  ,anti-depressant  anti-cancer , anti-bacterialanti-oxidant, anti- tumour,an insect deterrent andinsecticide. | (Li et al., 2018) |
|  | Betulinic acid | Triterpenoid | 64971 | *Ziziphus jujuba* | Act as anti-viral agent, inhibited the fungal growth,act as anti-inflammatory, anti-bacterial agent, anti-malarial, anti- HIV, anti- tumourAnd act as anti-feedant. | (Jasicka-Misiak et al., 2010) |
|  | Caffeic acid | Hydroxycinnsmic acid | 689043 | *Eucalyptus globulus* | Anti-viral,anti-fungal,anti-bacterial,anti-oxidant,anti- inflammatoryand insecticide. | (Sugiyama et al., 2016) |
|  | Camphor | terpenoid | 2537 | *Camphora officinarum* | Anti-viral, anti-fungal, anti-feedant, insecticide, an | (Tirillini et al., 1996) |
|  | Carvacrol | Monoterpene | 10364 | *Origanum vulgare* | Anti-fungal.anti-microbial, anti-cancer, insecticidal, anti-angiogenic, and anti-tumor activity | (Wijesundara et al., 2021) |
|  | Catechin | Flavonoids | 73160 | *Camellia sinensis* (Green tea) | Act as anti-viral, exhibit bioactivity against fungi. anti- cancer, anti- inflammatory,anti-bacterial and insecticidal agent. | (Yamaji, 2012) |
|  | Cinnamaldehyde | Phenylpropanoid | 637511 | *Cinnamomum cassia* | Anti-oxidant, anti-viral, anti-bacterial, anti-feedant, anti-inflammatory, anti-fungal and anti-cancer | (Wei et al., 2020) |
|  | Cucurbitacin E | Triterpenes | 5281319 | *Cucurbita foetidissima* | Act as anti-viral, anti-bacterial, anti-fungal, anti-inflammatory, hepatoprotective and anti-cancer. | (Martin & Schroder, 2000) |
|  | Curcumin | Phenolics | 969516 | *Curcuma longa* | Anti-fungal action against numerous fungi that are responsible for a variety of infections, prevent cancer, Modulate excitotoxicity,modulate neuroplasticity | (Hussain et al., 2022) |
|  | D-Limonene | monoterpene | 440917 | *Citrus sinensis* | Anti-viral, Anti-bacterial, Anti-fungal, insecticidal activity etc. | (Yu et al., 2022) |
|  | Ellagic Acid | Phenolics | 5281855 | *Punica granatum* | Anti-fungal activity.  anti-oxidant, anti-bacterial, and anti-inflammatory activities | (Li et al., 2015) |
|  | Emodin | Polyphenolic | 3220 | *Avicennia marina*(Grey mangrove) | Act against Hepatitis B virus,(inhibits HBV anti-gen)anti-bacterial, anti-ulcerogenic, anti-inflammatory, and anti-cancer effects, insecticide and anti-feedant. | (Kong et al., 2009) |
|  | Eucalyptol | Monoterpene | 2758 | *Eucalyptus obliqua* | anti-inflammatory, anti-bacterial, anti-viral, anti-fungal, anti-inflammatory and insect-repellent | (Sadlon & Lamson, 2010) |
|  | Ferulic acid | Phenolics | 445858 | *Oryza sativa* | Anti-fungal effects , anti-oxidant, anti-inflammatory, anti-microbial, anti-allergic, hepatoprotective, anti-carcinogenic, anti-thrombotic | (Khatkar et al., 2015) |
|  | Gallic acid | Phenolic acids | 370 | *Vitis vinifera, Fragaria ananassa, Vaccinium spp., Rubus spp* | Anti-bacterial, anti-fungal, anti-viral, anti- inflammatory, anti-cancer, anti-diabetic effects, insecticidal properties. | (Yang et al., 2020) |
|  | Geraniol | Monoterpene | 637566 | *Cymbopogon citratus* | Invitro anti-fungal potential against strains of C. albicans,anti-viral, anti-inflammatory, insecticide, anti-oxidant, anti-cancer | (Leite et al., 2015) |
|  | Hecogenin | Steroidal saponins | 91453 | *Agave sisalana* | Anti-inflammatory, anti-oxidant, anti-fungal, hypotensive, anti-hyperalgesic and anti-nociceptive | (Yang et al., 2006) |
|  | Hesperidin | flavonoid | 10621 | *Citrus auranti-um* | Anti-cancer, anti-fungal | (Ullah et al., 2020) |
|  | Kaempferol | flavonoid | 5280863 | *Camellia sinensis* | Anti-oxidant, anti-inflammatory, anti-microbial, cardiovascular, and neuroprotective | (Yang et al., 2006) |
|  | Luteolin | flavonoid | 5280445 | *Reseda luteola* | Anti-cancer, anti-fungal | (Ullah et al., 2020) |
|  | Matrine | Alkaloid | 91466 | *Sophora flavescens* | Anti-fungal,Cardiovascular protection,Anti-tumor,Anti-inflammatory,Immunosuppression,Anti-epileptic effects,Anti-nociceptive effects. | (Pan et al., 2019) |
|  | Naringenin | Flavonoid | 439246 | *Citrus sinensis*( Orange) | Anti-fungal, act as anti-viral, and insecticidal agent. | (Duda-Madej et al., 2022) |
|  | Paeonol | phenol | 11092 | *Paeonia suffruticosa* | Anti-bacterial, anti-fungal, insecticidal, anti-viral, anti-inflammatory, anti-pyretic analgesic, anti-oxidant, anti-cancer etc. | (Qian et al., 2022) |
|  | Quercetin | Flavonoid | 5280343 | *Allium cepa* (*onion)* | Anti-fungal potentials against Aspergillus fumigatus and Aspergillus niger, Act as anti-viral,(inhibit viral replication) anti-cancer, anti- Aphid or insecticidal agent | (Nguyen & Bhattacharya, 2022) |
|  | Resveratrol | Stilbenoid | 445154 | *Vitis amurensis* | Anti-feedant, potent anti-fungal activity against human pathogenic fungi, anti-oxidant, anti-tumor, anti-oxidant, anti-viral, and phytoestrogenic | (Jung et al., 2005) |
|  | Rosmarinic acid | Phenolics | 5281792 | *Rosmarinus officinalis L* | Anti-microbial, anti-fungal, anti-protozoal and anti-tumor activity. | (Bittner Fialová et al., 2019) |
|  | Rutin | Flavonoids | 5280805 |  | Act as anti-viral having strong anti-fungal action against pathogens, inhibit viral replication and insecticidal agent. | (Oliveira et al., 2016) |
|  | Thymol | Monoterpenoid phenol derivative | 6989 | *Thymus vulgaris* | Effective anti-viral agent,( inhibition of the virus-cell membrane fusion and viral assembly inside cells),anti-bacterial ,anti-parasitics, anti- fungal, anti-biofilms, anti-inflammatory, anti-diabetic, anti-rheumatic, ,anti-neurodegenerativ, insecticidal and anti-feedant against insect. | (Shcherbakova et al., 2021) |
|  | Thymoquinone | flavonoid | 10281 | *Nigella sativa* | Anti-oxidant, anti-fungal, insecticidal, anti-bacterial, anti-neoplastic, hepatoprotective, and anti-inflammatory activities | (Almshawit & Macreadie, 2017) |
|  | Trigonelline | Alkaloid | 5570 | *Trigonella foenum-graecum* | Anti-fungal activity, Hypoglycemic activity,Hypolipidemic activity,Neuroprotective activity,Anti-migraine activity,Sedative activity,Memory-improving activity,Anti-bacterial activity,Anti-viral activity. | (Almeida et al., 2006) |
|  | Turmerone | Sesquiterpene | 14367555 | *Curcuma longa* | Anti-inflammatory, immunomodulatory, anti-proliferative, and anti-fungal | (Obulesu, 2021) |
|  | Umbelliferone | Furanocoumarin | 5281426 | *Ficus septica* | Anti-oxidant, anti-cancer, anti-fungal | (Hirasawa & Takada, 2004) |

**References**

Almeida, A. A. P., Farah, A., Silva, D. A., Nunan, E. A., & Glória, M. B. A. (2006). Anti-bacterial activity of coffee extracts and selected coffee chemical compounds against enterobacteria. *Journal of agricultural and food chemistry*, *54*(23), 8738-8743.

Almshawit, H., & Macreadie, I. (2017). Fungicidal effect of thymoquinone involves generation of oxidative stress in Candida glabrata. *Microbiological Research*, *195*, 81-88. https://doi.org/https://doi.org/10.1016/j.micres.2016.11.008

Bittner Fialová, S., Kello, M., Čoma, M., Slobodníková, L., Drobná, E., Holková, I., Garajová, M., Mrva, M., Zachar, V., & Lukáč, M. (2019). Derivatization of Rosmarinic Acid Enhances its in vitro Anti-tumor, Anti-microbial and Anti-protozoal Properties. *Molecules*, *24*(6). https://doi.org/10.3390/molecules24061078

Castro, A., Lemos, C., Falcão, A., Fernandes, A. S., Glass, N. L., & Videira, A. (2010). Rotenone enhances the anti-fungal properties of staurosporine. *Eukaryot Cell*, *9*(6), 906-914. https://doi.org/10.1128/ec.00003-10

Duda-Madej, A., Stecko, J., Sobieraj, J., Szymańska, N., & Kozłowska, J. (2022). Naringenin and Its Derivatives-Health-Promoting Phytobiotic against Resistant Bacteria and Fungi in Humans. *Anti-biotics (Basel)*, *11*(11). https://doi.org/10.3390/anti-biotics11111628

Hirasawa, M., & Takada, K. (2004). Multiple effects of green tea catechin on the anti-fungal activity of anti-mycotics against Candida albicans. *Journal of Anti-microbial Chemotherapy*, *53*(2), 225-229.

Hussain, Y., Alam, W., Ullah, H., Dacrema, M., Daglia, M., Khan, H., & Arciola, C. R. (2022). Anti-microbial Potential of Curcumin: Therapeutic Potential and Challenges to Clinical Applications. *Anti-biotics (Basel)*, *11*(3). https://doi.org/10.3390/anti-biotics11030322

Jasicka-Misiak, I., Lipok, J., Swider, I. A., & Kafarski, P. (2010). Possible fungistatic implications of betulin presence in betulaceae plants and their hymenochaetaceae parasitic fungi. *Z Naturforsch C J Biosci*, *65*(3-4), 201-206. https://doi.org/10.1515/znc-2010-3-406

Jung, H. J., Hwang, I. A., Sung, W. S., Kang, H., Kang, B. S., Seu, Y. B., & Lee, D. G. (2005). Fungicidal effect of resveratrol on human infectious fungi. *Arch Pharm Res*, *28*(5), 557-560. https://doi.org/10.1007/bf02977758

Khatkar, A., Nanda, A., Kumar, P., & Narasimhan, B. (2015). Synthesis and anti-microbial evaluation of ferulic acid derivatives. *Research on Chemical Intermediates*, *41*(1), 299-309. https://doi.org/10.1007/s11164-013-1192-2

Kong, W. J., Wang, J. B., Jin, C., Zhao, Y. L., Dai, C. M., Xiao, X. H., & Li, Z. L. (2009). Effect of emodin on Candida albicans growth investigated by microcalorimetry combined with chemometric analysis. *Applied Microbiology and Biotechnology*, *83*(6), 1183-1190. https://doi.org/10.1007/s00253-009-2054-0

Leite, M. C., de Brito Bezerra, A. P., de Sousa, J. P., & de Oliveira Lima, E. (2015). Investigating the anti-fungal activity and mechanism(s) of geraniol against Candida albicans strains. *Med Mycol*, *53*(3), 275-284. https://doi.org/10.1093/mmy/myu078

Li, Y., Yin, Y. M., Wang, X. Y., Wu, H., & Ge, X. Z. (2018). Evaluation of berberine as a natural fungicide: biodegradation and anti-microbial mechanism. *J Asian Nat Prod Res*, *20*(2), 148-162. https://doi.org/10.1080/10286020.2017.1329300

Li, Z., Li, Z., Yang, J., Lu, C., Li, Y., Luo, Y., Cong, F., Shi, R., Wang, Z., Chen, H., Li, X., Yang, J., & Ye, F. (2022). Allicin shows anti-fungal efficacy against Cryptococcus neoformans by blocking the fungal cell membrane. *Front Microbiol*, *13*, 1012516. https://doi.org/10.3389/fmicb.2022.1012516

Li, Z. J., Guo, X., Dawuti, G., & Aibai, S. (2015). Anti-fungal Activity of Ellagic Acid In Vitro and In Vivo. *Phytother Res*, *29*(7), 1019-1025. https://doi.org/10.1002/ptr.5340

Martin, P., & Schroder, R. (2000). The Effect of Cucurbitacin E Glycoside, a Feeding Stimulant for Corn Rootworm, on Biocontrol Fungi: Beauveria bassiana and Metarhizium anisopliae. *Biocontrol Science and Technology*, *10*, 315-320. https://doi.org/10.1080/09583150050044583

Nguyen, T. L. A., & Bhattacharya, D. (2022). Anti-microbial Activity of Quercetin: An Approach to Its Mechanistic Principle. *Molecules*, *27*(8), 2494. https://www.mdpi.com/1420-3049/27/8/2494

Obulesu, M. (2021). Health benefits of turmeric: Emphasis on anti-cancer activity. *Journal: Turmeric and Curcumin for Neurodegenerative Diseases*, 3-18.

Oliveira, V. M., Carraro, E., Auler, M. E., & Khalil, N. M. (2016). Quercetin and rutin as potential agents anti-fungal against <i>Cryptococcus</i> spp. *Brazilian Journal of Biology*, *76*.

Pan, J., Hao, X., Yao, H., Ge, K., Ma, L., & Ma, W. (2019). Matrine inhibits mycelia growth of Botryosphaeria dothidea by affecting membrane permeability. *Journal of Forestry Research*, *30*(3), 1105-1113. https://doi.org/10.1007/s11676-019-00883-3

Qian, W., Li, X., Liu, Q., Lu, J., Wang, T., & Zhang, Q. (2022). Anti-fungal and Anti-biofilm Efficacy of Paeonol Treatment Against Biofilms Comprising Candida albicans and/or Cryptococcus neoformans [Original Research]. *Frontiers in Cellular and Infection Microbiology*, *12*. https://doi.org/10.3389/fcimb.2022.884793

Sadlon, A. E., & Lamson, D. W. (2010). Immune-modifying and anti-microbial effects of Eucalyptus oil and simple inhalation devices. *Altern Med Rev*, *15*(1), 33-47.

Shcherbakova, L., Mikityuk, O., Arslanova, L., Stakheev, A., Erokhin, D., Zavriev, S., & Dzhavakhiya, V. (2021). Studying the Ability of Thymol to Improve Fungicidal Effects of Tebuconazole and Difenoconazole Against Some Plant Pathogenic Fungi in Seed or Foliar Treatments [Original Research]. *Frontiers in Microbiology*, *12*. https://doi.org/10.3389/fmicb.2021.629429

Sugiyama, A., Sano, C. M., Yazaki, K., & Sano, H. (2016). Caffeine fostering of mycoparasitic fungi against phytopathogens. *Plant Signal Behav*, *11*(1), e1113362. https://doi.org/10.1080/15592324.2015.1113362

Thomaz, L., Apitz-Castro, R., Marques, A. F., Travassos, L. R., & Taborda, C. P. (2008). Experimental paracoccidioidomycosis: alternative therapy with ajoene, compound from Allium sativum, associated with sulfamethoxazole/trimethoprim. *Med Mycol*, *46*(2), 113-118. https://doi.org/10.1080/13693780701651681

Tirillini, B., Velasquez, E. R., & Pellegrino, R. (1996). Chemical composition and anti-microbial activity of essential oil of Piper angustifolium. *Planta Med*, *62*(4), 372-373. https://doi.org/10.1055/s-2006-957911

Ullah, A., Munir, S., Badshah, S. L., Khan, N., Ghani, L., Poulson, B. G., Emwas, A.-H., & Jaremko, M. (2020). Important Flavonoids and Their Role as a Therapeutic Agent. *Molecules*, *25*(22), 5243. https://www.mdpi.com/1420-3049/25/22/5243

Wei, J., Bi, Y., Xue, H., Wang, Y., Zong, Y., & Prusky, D. (2020). Anti-fungal activity of cinnamaldehyde against Fusarium sambucinum involves inhibition of ergosterol biosynthesis. *J Appl Microbiol*, *129*(2), 256-265. https://doi.org/10.1111/jam.14601

Wijesundara, N. M., Lee, S. F., Cheng, Z., Davidson, R., & Rupasinghe, H. P. V. (2021). Carvacrol exhibits rapid bactericidal activity against Streptococcus pyogenes through cell membrane damage. *Scientific Reports*, *11*(1), 1487. https://doi.org/10.1038/s41598-020-79713-0

Yamaji, K. (2012). The role of catechin and epicatechin in chemical defense against damping‐off fungi of current‐year Fagus crenata seedlings in natural forest. *Forest pathology*, *v. 42*(no. 1), pp. 1-7-2012 v.2042 no.2011. https://doi.org/10.1111/j.1439-0329.2010.00709.x

Yang, C.-R., Zhang, Y., Jacob, M. R., Khan, S. I., Zhang, Y.-J., & Li, X.-C. (2006). Anti-fungal activity of C-27 steroidal saponins. *Anti-microbial Agents and Chemotherapy*, *50*(5), 1710-1714.

Yang, K., Zhang, L., Liao, P., Xiao, Z., Zhang, F., Sindaye, D., Xin, Z., Tan, C., Deng, J., Yin, Y., & Deng, B. (2020). Impact of Gallic Acid on Gut Health: Focus on the Gut Microbiome, Immune Response, and Mechanisms of Action. *Front Immunol*, *11*, 580208. https://doi.org/10.3389/fimmu.2020.580208

Yu, H., Lin, Z.-X., Xiang, W.-L., Huang, M., Tang, J., Lu, Y., Zhao, Q.-H., Zhang, Q., Rao, Y., & Liu, L. (2022). Anti-fungal activity and mechanism of d-limonene against foodborne opportunistic pathogen Candida tropicalis. *LWT*, *159*, 113144. https://doi.org/https://doi.org/10.1016/j.lwt.2022.113144
