# Supplementary material for: Exploring effector protein dynamics and natural fungicidal potential in rice blast pathogen Magnaporthe oryzae
Source: PLoS One. 2025 Jan 24;20(1):e0307352. doi: 10.1371/journal.pone.0307352 (PMC11761166; doi:10.1371/journal.pone.0307352)
Supplement: S2 Table — (DOCX) [file pone.0307352.s002.docx]

**Supplementary Table S2**: Result of Molecular Docking of Effector Proteins and Ligands

| **Ligand** | **Binding Affinity** | **RMSD/UB** | **RMSD/LB** |
| --- | --- | --- | --- |
| Apikl2a__Hecogenin_uff_E=836.11 | -8.8 | 0 | 0 |
| Apikl2a__ Rosmarinic_acid _uff_E=293.67 | -8 | 0 | 0 |
| Apikl2a__Rutin_uff_E=751.29 | -7.7 | 0 | 0 |
| Apikl2a__Rotenone_uff_E=627.57 | -7.6 | 0 | 0 |
| Apikl2a__Luteolin_uff_E=241.99 | -7.5 | 0 | 0 |
| Apikl2a__CucurbitacinE_uff_E=864.84 | -7.4 | 0 | 0 |
| Apikl2a__Hesperedin_uff_E=589.61 | -7.4 | 0 | 0 |
| Apikl2a__Betulinicacid_uff_E=790.90 | -7.3 | 0 | 0 |
| Apikl2a__Quercetin_uff_E=379.98 | -7.3 | 0 | 0 |
| strobulirin | -7.2 |  |  |
| Apikl2a__Kaempferol_uff_E=362.50 | -7.2 | 0 | 0 |
| Apikl2a__Naringenin_uff_E=195.82 | -7.1 | 0 | 0 |
| Apikl2a__Catechin_uff_E=205.12 | -7 | 0 | 0 |
| Apikl2a__Resveratrol_uff_E=172.23 | -7 | 0 | 0 |
| Apikl2a__Berberine_uff_E=579.71 | -6.9 | 0 | 0 |
| Apikl2a__Curcumin_uff_E=1321.66 | -6.8 | 0 | 0 |
| Apikl2a__Emodin_uff_E=196.49 | -6.8 | 0 | 0 |
| Apikl2a__Ellagic_Acid_uff_E=215.94 | -6.7 | 0 | 0 |
| Apikl2a__Turmerone_uff_E=144.16 | -6.5 | 0 | 0 |
| Apikl2a__Matrine_uff_E=243.55 | -6.3 | 0 | 0 |
| Apikl2a__Caffeicacid_uff_E=98.70 | -6.1 | 0 | 0 |
| Apikl2a__Ferulic_acid_uff_E=470.76 | -5.9 | 0 | 0 |
| Apikl2a__Umbelliferone_uff_E=96.98 | -5.7 | 0 | 0 |
| Apikl2a__Carvacrol_uff_E=78.47 | -5.5 | 0 | 0 |
| Apikl2a__Thymol_uff_E=95.99 | -5.5 | 0 | 0 |
| Apikl2a__Thymoquinone_uff_E=62.47 | -5.5 | 0 | 0 |
| Apikl2a__D-Limonene_uff_E=101.93 | -5.4 | 0 | 0 |
| Apikl2a__Paeonol_uff_E=127.65 | -5.4 | 0 | 0 |
| Apikl2a__Gallicacid_uff_E=77.81 | -5.3 | 0 | 0 |
| Apikl2a__Geraniol_uff_E=109.74 | -5.3 | 0 | 0 |
| Apikl2a__Cinnamaldehyde_uff_E=76.51 | -5.1 | 0 | 0 |
| Apikl2a__Trigonelline_uff_E=412.52 | -5.1 | 0 | 0 |
| Apikl2a__Camphor_uff_E=400.52 | -5 | 0 | 0 |
| Apikl2a__Eucalyptol_uff_E=344.57 | -4.7 | 0 | 0 |
| Apikl2a__Ajoene_uff_E=148.82 | -4.2 | 0 | 0 |
| Apikl2a__Allicin_uff_E=111.53 | -4.1 | 0 | 0 |

| **Ligand** | **Binding Affinity** | **RMSD/UB** | **RMSD/LB** |
| --- | --- | --- | --- |
| APikL2F_Hecogenin_uff_E=836.11 | -8.5 | 0 | 0 |
| APikL2F_Rutin_uff_E=751.29 | -8.2 | 0 | 0 |
| APikL2F_Hesperedin_uff_E=589.61 | -8 | 0 | 0 |
| APikL2F_Betulinicacid_uff_E=790.90 | -7.7 | 0 | 0 |
| APikL2F_Quercetin_uff_E=379.98 | -7.4 | 0 | 0 |
| APikL2F_Luteolin_uff_E=241.99 | -7.3 | 0 | 0 |
| APikL2F_Rotenone_uff_E=627.57 | -7.3 | 0 | 0 |
| APikL2F_CucurbitacinE_uff_E=864.84 | -7.2 | 0 | 0 |
| APikL2F_Naringenin_uff_E=195.82 | -7.2 | 0 | 0 |
| APikL2F_Ellagic_Acid_uff_E=215.94 | -7.1 | 0 | 0 |
| APikL2F_Emodin_uff_E=196.49 | -7 | 0 | 0 |
| APikL2F_Berberine_uff_E=579.71 | -6.9 | 0 | 0 |
| APikL2F_Kaempferol_uff_E=362.50 | -6.9 | 0 | 0 |
| APikL2F_Catechin_uff_E=205.12 | -6.8 | 0 | 0 |
| APikL2F_Resveratrol_uff_E=172.23 | -6.6 | 0 | 0 |
| APikL2F_Rosmarinic_acid_uff_E=293.67 | -6.4 | 0 | 0 |
| APik2f_Strobulirin(control) | -6.5 |  |  |
| APikL2F_Curcumin_uff_E=1321.66 | -6.1 | 0 | 0 |
| APikL2F_Matrine_uff_E=243.55 | -5.8 | 0 | 0 |
| APikL2F_Thymoquinone_uff_E=62.47 | -5.6 | 0 | 0 |
| APikL2F_Umbelliferone_uff_E=96.98 | -5.6 | 0 | 0 |
| APikL2F_Ferulic_acid_uff_E=470.76 | -5.4 | 0 | 0 |
| APikL2F_Turmerone_uff_E=144.16 | -5.4 | 0 | 0 |
| APikL2F_Caffeicacid_uff_E=98.70 | -5.3 | 0 | 0 |
| APikL2F_Carvacrol_uff_E=78.47 | -5.3 | 0 | 0 |
| APikL2F_Gallicacid_uff_E=77.81 | -5.2 | 0 | 0 |
| APikL2F_Thymol_uff_E=95.99 | -5.2 | 0 | 0 |
| APikL2F_Cinnamaldehyde_uff_E=76.51 | -5.1 | 0 | 0 |
| APikL2F_D-Limonene_uff_E=101.93 | -4.9 | 0 | 0 |
| APikL2F_Paeonol_uff_E=127.65 | -4.9 | 0 | 0 |
| APikL2F_Camphor_uff_E=400.52 | -4.7 | 0 | 0 |
| APikL2F_Geraniol_uff_E=109.74 | -4.7 | 0 | 0 |
| APikL2F_Eucalyptol_uff_E=344.57 | -4.5 | 0 | 0 |
| APikL2F_Trigonelline_uff_E=412.52 | -4.5 | 0 | 0 |
| APikL2F_Ajoene_uff_E=148.82 | -3.8 | 0 | 0 |
| APikL2F_Allicin_uff_E=111.53 | -3.2 | 0 | 0 |

| **Ligand** | **Binding Affinity** | **RMSD/UB** | **RMSD/LB** |
| --- | --- | --- | --- |
| AVR-Pia_Hecogenin_uff_E=836.11 | -7.7 | 0 | 0 |
| AVR-Pia_Hesperedin_uff_E=589.61 | -7 | 0 | 0 |
| AVR-Pia_Luteolin_uff_E=241.99 | -7 | 0 | 0 |
| AVR-Pia_Rotenone_uff_E=627.57 | -7 | 0 | 0 |
| AVR-Pia_Rosmarinic_acid_uff_E=293.67 | -6.9 | 0 | 0 |
| AVR-Pia_Berberine_uff_E=579.71 | -6.8 | 0 | 0 |
| AVR-Pia_CucurbitacinE_uff_E=864.84 | -6.7 | 0 | 0 |
| AVR-Pia_Catechin_uff_E=205.12 | -6.6 | 0 | 0 |
| AVR-Pia_Naringenin_uff_E=195.82 | -6.6 | 0 | 0 |
| AVR-Pia_Quercetin_uff_E=379.98 | -6.6 | 0 | 0 |
| AVR-Pia_Rutin_uff_E=751.29 | -6.5 | 0 | 0 |
| AVR-Pia_Resveratrol_uff_E=172.23 | -6.4 | 0 | 0 |
| AVR-Pia_Kaempferol_uff_E=362.50 | -6.3 | 0 | 0 |
| AVR-Pia_Strobulirin(control) | -6.2 |  |  |
| AVR-Pia_Betulinicacid_uff_E=790.90 | -6.2 | 0 | 0 |
| AVR-Pia_Curcumin_uff_E=1321.66 | -6.2 | 0 | 0 |
| AVR-Pia_Emodin_uff_E=196.49 | -6.2 | 0 | 0 |
| AVR-Pia_Ellagic_Acid_uff_E=215.94 | -6 | 0 | 0 |
| AVR-Pia_Matrine_uff_E=243.55 | -5.6 | 0 | 0 |
| AVR-Pia_Caffeicacid_uff_E=98.70 | -5.4 | 0 | 0 |
| AVR-Pia_Umbelliferone_uff_E=96.98 | -5.3 | 0 | 0 |
| AVR-Pia_Ferulic_acid_uff_E=470.76 | -5.2 | 0 | 0 |
| AVR-Pia_Thymol_uff_E=95.99 | -5.2 | 0 | 0 |
| AVR-Pia_Thymoquinone_uff_E=62.47 | -5.2 | 0 | 0 |
| AVR-Pia_Paeonol_uff_E=127.65 | -5.1 | 0 | 0 |
| AVR-Pia_Gallicacid_uff_E=77.81 | -4.9 | 0 | 0 |
| AVR-Pia_Turmerone_uff_E=144.16 | -4.9 | 0 | 0 |
| AVR-Pia_Carvacrol_uff_E=78.47 | -4.8 | 0 | 0 |
| AVR-Pia_Geraniol_uff_E=109.74 | -4.7 | 0 | 0 |
| AVR-Pia_Trigonelline_uff_E=412.52 | -4.6 | 0 | 0 |
| AVR-Pia_Eucalyptol_uff_E=344.57 | -4.4 | 0 | 0 |
| AVR-Pia_D-Limonene_uff_E=101.93 | -4.3 | 0 | 0 |
| AVR-Pia_Camphor_uff_E=400.52 | -4.2 | 0 | 0 |
| AVR-Pia_Cinnamaldehyde_uff_E=76.51 | -4.2 | 0 | 0 |
| AVR-Pia_Ajoene_uff_E=148.82 | -3.5 | 0 | 0 |
| AVR-Pia_Allicin_uff_E=111.53 | -3.1 | 0 | 0 |

| **Ligand** | **Binding Affinity** | **RMSD/UB** | **RMSD/LB** |
| --- | --- | --- | --- |
| AvrPib_Hesperedin_uff_E=589.61 | -8.1 | 0 | 0 |
| AvrPib_Betulinicacid_uff_E=790.90 | -7.9 | 0 | 0 |
| AvrPib_Hecogenin_uff_E=836.11 | -7.7 | 0 | 0 |
| AvrPib_Emodin_uff_E=196.49 | -7.6 | 0 | 0 |
| AvrPib_Quercetin_uff_E=379.98 | -7.6 | 0 | 0 |
| AvrPib_Rutin_uff_E=751.29 | -7.6 | 0 | 0 |
| AvrPib_CucurbitacinE_uff_E=864.84 | -7.5 | 0 | 0 |
| AvrPib_Ellagic_Acid_uff_E=215.94 | -7.4 | 0 | 0 |
| AvrPib_Kaempferol_uff_E=362.50 | -7.4 | 0 | 0 |
| AvrPib_Rosmarinic_acid_uff_E=293.67 | -7.4 | 0 | 0 |
| AvrPib_Berberine_uff_E=579.71 | -7.3 | 0 | 0 |
| AvrPib_Luteolin_uff_E=241.99 | -7.3 | 0 | 0 |
| AvrPib_Rotenone_uff_E=627.57 | -7.2 | 0 | 0 |
| strobulirin | -7.1 |  |  |
| AvrPib_Catechin_uff_E=205.12 | -7.1 | 0 | 0 |
| AvrPib_Naringenin_uff_E=195.82 | -6.9 | 0 | 0 |
| AvrPib_Curcumin_uff_E=1321.66 | -6.6 | 0 | 0 |
| AvrPib_Resveratrol_uff_E=172.23 | -6.3 | 0 | 0 |
| AvrPib_Umbelliferone_uff_E=96.98 | -6.2 | 0 | 0 |
| AvrPib_Carvacrol_uff_E=78.47 | -6.1 | 0 | 0 |
| AvrPib_Matrine_uff_E=243.55 | -6.1 | 0 | 0 |
| AvrPib_Gallicacid_uff_E=77.81 | -6 | 0 | 0 |
| AvrPib_Caffeicacid_uff_E=98.70 | -5.9 | 0 | 0 |
| AvrPib_Turmerone_uff_E=144.16 | -5.9 | 0 | 0 |
| AvrPib_Ferulic_acid_uff_E=470.76 | -5.7 | 0 | 0 |
| AvrPib_Thymoquinone_uff_E=62.47 | -5.6 | 0 | 0 |
| AvrPib_D-Limonene_uff_E=101.93 | -5.5 | 0 | 0 |
| AvrPib_Thymol_uff_E=95.99 | -5.5 | 0 | 0 |
| AvrPib_Cinnamaldehyde_uff_E=76.51 | -5.4 | 0 | 0 |
| AvrPib_Paeonol_uff_E=127.65 | -5.3 | 0 | 0 |
| AvrPib_Geraniol_uff_E=109.74 | -5.2 | 0 | 0 |
| AvrPib_Trigonelline_uff_E=412.52 | -5.2 | 0 | 0 |
| AvrPib_Eucalyptol_uff_E=344.57 | -5 | 0 | 0 |
| AvrPib_Camphor_uff_E=400.52 | -4.8 | 0 | 0 |
| AvrPib_Ajoene_uff_E=148.82 | -3.9 | 0 | 0 |
| AvrPib_Allicin_uff_E=111.53 | -3.8 | 0 | 0 |

| **Ligand** | **Binding Affinity** | **RMSD/UB** | **RMSD/LB** |
| --- | --- | --- | --- |
| AVR-Pii_Rutin_uff_E=751.29 | -6.2 | 0 | 0 |
| AVR-Pii_Hecogenin_uff_E=836.11 | -6.1 | 0 | 0 |
| AVR-Pii_Rosmarinic_acid_uff_E=293.67 | -6.1 | 0 | 0 |
| AVR-Pii_Rotenone_uff_E=627.57 | -5.8 | 0 | 0 |
| AVR-Pii_Curcumin_uff_E=1321.66 | -5.7 | 0 | 0 |
| AVR-Pii_Hesperedin_uff_E=589.61 | -5.7 | 0 | 0 |
| AVR-Pii_Ellagic_Acid_uff_E=215.94 | -5.6 | 0 | 0 |
| AVR-Pii_Strobulirin(control) | -5.4 |  |  |
| AVR-Pii_Luteolin_uff_E=241.99 | -5.5 | 0 | 0 |
| AVR-Pii_Catechin_uff_E=205.12 | -5.4 | 0 | 0 |
| AVR-Pii_CucurbitacinE_uff_E=864.84 | -5.4 | 0 | 0 |
| AVR-Pii_Kaempferol_uff_E=362.50 | -5.4 | 0 | 0 |
| AVR-Pii_Berberine_uff_E=579.71 | -5.3 | 0 | 0 |
| AVR-Pii_Quercetin_uff_E=379.98 | -5.2 | 0 | 0 |
| AVR-Pii_Emodin_uff_E=196.49 | -5.1 | 0 | 0 |
| AVR-Pii_Naringenin_uff_E=195.82 | -5.1 | 0 | 0 |
| AVR-Pii_Betulinicacid_uff_E=790.90 | -5 | 0 | 0 |
| AVR-Pii_Matrine_uff_E=243.55 | -4.9 | 0 | 0 |
| AVR-Pii_Resveratrol_uff_E=172.23 | -4.9 | 0 | 0 |
| AVR-Pii_Umbelliferone_uff_E=96.98 | -4.3 | 0 | 0 |
| AVR-Pii_Caffeicacid_uff_E=98.70 | -4.2 | 0 | 0 |
| AVR-Pii_Gallicacid_uff_E=77.81 | -4.2 | 0 | 0 |
| AVR-Pii_Ferulic_acid_uff_E=470.76 | -4.1 | 0 | 0 |
| AVR-Pii_Turmerone_uff_E=144.16 | -4.1 | 0 | 0 |
| AVR-Pii_Carvacrol_uff_E=78.47 | -3.9 | 0 | 0 |
| AVR-Pii_Paeonol_uff_E=127.65 | -3.9 | 0 | 0 |
| AVR-Pii_Thymol_uff_E=95.99 | -3.8 | 0 | 0 |
| AVR-Pii_Thymoquinone_uff_E=62.47 | -3.8 | 0 | 0 |
| AVR-Pii_Cinnamaldehyde_uff_E=76.51 | -3.6 | 0 | 0 |
| AVR-Pii_Geraniol_uff_E=109.74 | -3.6 | 0 | 0 |
| AVR-Pii_Trigonelline_uff_E=412.52 | -3.6 | 0 | 0 |
| AVR-Pii_Camphor_uff_E=400.52 | -3.5 | 0 | 0 |
| AVR-Pii_D-Limonene_uff_E=101.93 | -3.5 | 0 | 0 |
| AVR-Pii_Eucalyptol_uff_E=344.57 | -3.2 | 0 | 0 |
| AVR-Pii_Ajoene_uff_E=148.82 | -3 | 0 | 0 |
| AVR-Pii_Allicin_uff_E=111.53 | -2.4 | 0 | 0 |

| **Ligand** | **Binding Affinity** | **RMSD/UB** | **RMSD/LB** |
| --- | --- | --- | --- |
| AVR-PikA_Betulinicacid_uff_E=790.90 | -8 | 0 | 0 |
| AVR-PikA_Hecogenin_uff_E=836.11 | -7.5 | 0 | 0 |
| AVR-PikA_Rutin_uff_E=751.29 | -7.5 | 0 | 0 |
| AVR-PikA_CucurbitacinE_uff_E=864.84 | -7.3 | 0 | 0 |
| AVR-PikA_Hesperedin_uff_E=589.61 | -7.1 | 0 | 0 |
| AVR-PikA_Rosmarinic_acid_uff_E=293.67 | -6.8 | 0 | 0 |
| AVR-PikA_Ellagic_Acid_uff_E=215.94 | -6.7 | 0 | 0 |
| AVR-PikA_Quercetin_uff_E=379.98 | -6.6 | 0 | 0 |
| AVR-PikA_Rotenone_uff_E=627.57 | -6.6 | 0 | 0 |
| AR-pikA_Strobulirin(control) | -6.5 |  |  |
| AVR-PikA_Berberine_uff_E=579.71 | -6.5 | 0 | 0 |
| AVR-PikA_Curcumin_uff_E=1321.66 | -6.5 | 0 | 0 |
| AVR-PikA_Luteolin_uff_E=241.99 | -6.5 | 0 | 0 |
| AVR-PikA_Kaempferol_uff_E=362.50 | -6.4 | 0 | 0 |
| AVR-PikA_Catechin_uff_E=205.12 | -6.2 | 0 | 0 |
| AVR-PikA_Emodin_uff_E=196.49 | -6.2 | 0 | 0 |
| AVR-PikA_Naringenin_uff_E=195.82 | -6.2 | 0 | 0 |
| AVR-PikA_Resveratrol_uff_E=172.23 | -6 | 0 | 0 |
| AVR-PikA_Umbelliferone_uff_E=96.98 | -5.6 | 0 | 0 |
| AVR-PikA_Caffeicacid_uff_E=98.70 | -5.5 | 0 | 0 |
| AVR-PikA_Matrine_uff_E=243.55 | -5.5 | 0 | 0 |
| AVR-PikA_Turmerone_uff_E=144.16 | -5.4 | 0 | 0 |
| AVR-PikA_Carvacrol_uff_E=78.47 | -5.3 | 0 | 0 |
| AVR-PikA_Thymoquinone_uff_E=62.47 | -5.2 | 0 | 0 |
| AVR-PikA_D-Limonene_uff_E=101.93 | -5.1 | 0 | 0 |
| AVR-PikA_Ferulic_acid_uff_E=470.76 | -5.1 | 0 | 0 |
| AVR-PikA_Cinnamaldehyde_uff_E=76.51 | -5 | 0 | 0 |
| AVR-PikA_Thymol_uff_E=95.99 | -5 | 0 | 0 |
| AVR-PikA_Camphor_uff_E=400.52 | -4.9 | 0 | 0 |
| AVR-PikA_Eucalyptol_uff_E=344.57 | -4.8 | 0 | 0 |
| AVR-PikA_Geraniol_uff_E=109.74 | -4.8 | 0 | 0 |
| AVR-PikA_Paeonol_uff_E=127.65 | -4.5 | 0 | 0 |
| AVR-PikA_Gallicacid_uff_E=77.81 | -4.4 | 0 | 0 |
| AVR-PikA_Trigonelline_uff_E=412.52 | -4.1 | 0 | 0 |
| AVR-PikA_Ajoene_uff_E=148.82 | -3.6 | 0 | 0 |
| AVR-PikA_Allicin_uff_E=111.53 | -3.1 | 0 | 0 |

| **Ligand** | **Binding Affinity** | **RMSD/UB** | **RMSD/LB** |
| --- | --- | --- | --- |
| AVR-PikC_Hecogenin_uff_E=836.11 | -8.3 | 0 | 0 |
| AVR-PikC_Hesperedin_uff_E=589.61 | -7.7 | 0 | 0 |
| AVR-PikC_Quercetin_uff_E=379.98 | -7.7 | 0 | 0 |
| AVR-PikC_Rutin_uff_E=751.29 | -7.7 | 0 | 0 |
| AVR-PikC_Luteolin_uff_E=241.99 | -7.5 | 0 | 0 |
| AVR-PikC_Kaempferol_uff_E=362.50 | -7.4 | 0 | 0 |
| AVR-PikC_Naringenin_uff_E=195.82 | -7.3 | 0 | 0 |
| AVR-PikC_Betulinicacid_uff_E=790.90 | -7.2 | 0 | 0 |
| AVR-PikC_CucurbitacinE_uff_E=864.84 | -7.2 | 0 | 0 |
| AVR-PikC_Ellagic_Acid_uff_E=215.94 | -7.1 | 0 | 0 |
| AVR-PikC_Catechin_uff_E=205.12 | -6.9 | 0 | 0 |
| AVR-PikC_Berberine_uff_E=579.71 | -6.8 | 0 | 0 |
| AVR-PikC_Rotenone_uff_E=627.57 | -6.8 | 0 | 0 |
| AVR-PikC_Curcumin_uff_E=1321.66 | -6.7 | 0 | 0 |
| AVR-PikC_Rosmarinic_acid_uff_E=293.67 | -6.7 | 0 | 0 |
| AVR_PikC_Strobuilirin(control) | -6.6 |  |  |
| AVR-PikC_Emodin_uff_E=196.49 | -6.5 | 0 | 0 |
| AVR-PikC_Resveratrol_uff_E=172.23 | -6.3 | 0 | 0 |
| AVR-PikC_Umbelliferone_uff_E=96.98 | -5.8 | 0 | 0 |
| AVR-PikC_Caffeicacid_uff_E=98.70 | -5.5 | 0 | 0 |
| AVR-PikC_Carvacrol_uff_E=78.47 | -5.4 | 0 | 0 |
| AVR-PikC_Matrine_uff_E=243.55 | -5.4 | 0 | 0 |
| AVR-PikC_Turmerone_uff_E=144.16 | -5.4 | 0 | 0 |
| AVR-PikC_Ferulic_acid_uff_E=470.76 | -5.2 | 0 | 0 |
| AVR-PikC_Thymol_uff_E=95.99 | -5.2 | 0 | 0 |
| AVR-PikC_D-Limonene_uff_E=101.93 | -5.1 | 0 | 0 |
| AVR-PikC_Cinnamaldehyde_uff_E=76.51 | -5 | 0 | 0 |
| AVR-PikC_Eucalyptol_uff_E=344.57 | -5 | 0 | 0 |
| AVR-PikC_Thymoquinone_uff_E=62.47 | -5 | 0 | 0 |
| AVR-PikC_Camphor_uff_E=400.52 | -4.9 | 0 | 0 |
| AVR-PikC_Gallicacid_uff_E=77.81 | -4.9 | 0 | 0 |
| AVR-PikC_Geraniol_uff_E=109.74 | -4.8 | 0 | 0 |
| AVR-PikC_Paeonol_uff_E=127.65 | -4.8 | 0 | 0 |
| AVR-PikC_Trigonelline_uff_E=412.52 | -4.4 | 0 | 0 |
| AVR-PikC_Ajoene_uff_E=148.82 | -3.3 | 0 | 0 |
| AVR-PikC_Allicin_uff_E=111.53 | -2.9 | 0 | 0 |

| **Ligand** | **Binding Affinity** | **RMSD/UB** | **RMSD/LB** |
| --- | --- | --- | --- |
| AVR-PikD_Hecogenin_uff_E=836.11 | -7.8 | 0 | 0 |
| AVR-PikD_Hesperedin_uff_E=589.61 | -7.4 | 0 | 0 |
| AVR-PikD_Rutin_uff_E=751.29 | -7.3 | 0 | 0 |
| Strobullirin(control) | -6.8 |  |  |
| AVR-PikD_Rotenone_uff_E=627.57 | -6.9 | 0 | 0 |
| AVR-PikD_CucurbitacinE_uff_E=864.84 | -6.8 | 0 | 0 |
| AVR-PikD_Curcumin_uff_E=1321.66 | -6.8 | 0 | 0 |
| AVR-PikD_Betulinicacid_uff_E=790.90 | -6.7 | 0 | 0 |
| AVR-PikD_Rosmarinic_acid_uff_E=293.67 | -6.7 | 0 | 0 |
| AVR-PikD_Luteolin_uff_E=241.99 | -6.6 | 0 | 0 |
| AVR-PikD_Berberine_uff_E=579.71 | -6.5 | 0 | 0 |
| AVR-PikD_Ellagic_Acid_uff_E=215.94 | -6.5 | 0 | 0 |
| AVR-PikD_Quercetin_uff_E=379.98 | -6.4 | 0 | 0 |
| AVR-PikD_Catechin_uff_E=205.12 | -6.2 | 0 | 0 |
| AVR-PikD_Kaempferol_uff_E=362.50 | -6.2 | 0 | 0 |
| AVR-PikD_Resveratrol_uff_E=172.23 | -6.2 | 0 | 0 |
| AVR-PikD_Naringenin_uff_E=195.82 | -6.1 | 0 | 0 |
| AVR-PikD_Emodin_uff_E=196.49 | -6 | 0 | 0 |
| AVR-PikD_Matrine_uff_E=243.55 | -5.7 | 0 | 0 |
| AVR-PikD_Turmerone_uff_E=144.16 | -5.5 | 0 | 0 |
| AVR-PikD_Caffeicacid_uff_E=98.70 | -5.4 | 0 | 0 |
| AVR-PikD_Carvacrol_uff_E=78.47 | -5.4 | 0 | 0 |
| AVR-PikD_Umbelliferone_uff_E=96.98 | -5.4 | 0 | 0 |
| AVR-PikD_Cinnamaldehyde_uff_E=76.51 | -5.2 | 0 | 0 |
| AVR-PikD_Ferulic_acid_uff_E=470.76 | -5.2 | 0 | 0 |
| AVR-PikD_Geraniol_uff_E=109.74 | -5 | 0 | 0 |
| AVR-PikD_Thymol_uff_E=95.99 | -5 | 0 | 0 |
| AVR-PikD_D-Limonene_uff_E=101.93 | -4.9 | 0 | 0 |
| AVR-PikD_Thymoquinone_uff_E=62.47 | -4.8 | 0 | 0 |
| AVR-PikD_Camphor_uff_E=400.52 | -4.7 | 0 | 0 |
| AVR-PikD_Gallicacid_uff_E=77.81 | -4.7 | 0 | 0 |
| AVR-PikD_Eucalyptol_uff_E=344.57 | -4.6 | 0 | 0 |
| AVR-PikD_Paeonol_uff_E=127.65 | -4.4 | 0 | 0 |
| AVR-PikD_Trigonelline_uff_E=412.52 | -4.3 | 0 | 0 |
| AVR-PikD_Ajoene_uff_E=148.82 | -3.6 | 0 | 0 |
| AVR-PikD_Allicin_uff_E=111.53 | -3.1 | 0 | 0 |

| **Ligand** | **Binding Affinity** | **RMSD/UB** | **RMSD/LB** |
| --- | --- | --- | --- |
| AVR-PikE_Hecogenin_uff_E=836.11 | -7.2 | 0 | 0 |
| AVR-PikE_Rutin_uff_E=751.29 | -7 | 0 | 0 |
| AVR-PikE_Hesperedin_uff_E=589.61 | -6.8 | 0 | 0 |
| AVR-PikE_Betulinicacid_uff_E=790.90 | -6.5 | 0 | 0 |
| AVR-PikE_CucurbitacinE_uff_E=864.84 | -6.5 | 0 | 0 |
| AVR-PikE_Rotenone_uff_E=627.57 | -6.4 | 0 | 0 |
| AVR-PikE_Naringenin_uff_E=195.82 | -6.3 | 0 | 0 |
| AVR-PikE_Luteolin_uff_E=241.99 | -6.1 | 0 | 0 |
| AVR-PikE_Quercetin_uff_E=379.98 | -6.1 | 0 | 0 |
| AVR-PikE_Berberine_uff_E=579.71 | -6 | 0 | 0 |
| AVR-PikE_Catechin_uff_E=205.12 | -6 | 0 | 0 |
| AVR-PikE_Kaempferol_uff_E=362.50 | -6 | 0 | 0 |
| AVR-PikE_Rosmarinic_acid_uff_E=293.67 | -5.9 | 0 | 0 |
| AVR-PikE_Emodin_uff_E=196.49 | -5.8 | 0 | 0 |
| Strobulirin(control) | -5.7 |  |  |
| AVR-PikE_Ellagic_Acid_uff_E=215.94 | -5.7 | 0 | 0 |
| AVR-PikE_Curcumin_uff_E=1321.66 | -5.6 | 0 | 0 |
| AVR-PikE_Matrine_uff_E=243.55 | -5.4 | 0 | 0 |
| AVR-PikE_Resveratrol_uff_E=172.23 | -5.3 | 0 | 0 |
| AVR-PikE_Turmerone_uff_E=144.16 | -5.1 | 0 | 0 |
| AVR-PikE_Umbelliferone_uff_E=96.98 | -4.8 | 0 | 0 |
| AVR-PikE_Ferulic_acid_uff_E=470.76 | -4.6 | 0 | 0 |
| AVR-PikE_Gallicacid_uff_E=77.81 | -4.6 | 0 | 0 |
| AVR-PikE_Caffeicacid_uff_E=98.70 | -4.5 | 0 | 0 |
| AVR-PikE_Camphor_uff_E=400.52 | -4.5 | 0 | 0 |
| AVR-PikE_Carvacrol_uff_E=78.47 | -4.5 | 0 | 0 |
| AVR-PikE_D-Limonene_uff_E=101.93 | -4.4 | 0 | 0 |
| AVR-PikE_Paeonol_uff_E=127.65 | -4.4 | 0 | 0 |
| AVR-PikE_Thymol_uff_E=95.99 | -4.4 | 0 | 0 |
| AVR-PikE_Thymoquinone_uff_E=62.47 | -4.4 | 0 | 0 |
| AVR-PikE_Eucalyptol_uff_E=344.57 | -4.3 | 0 | 0 |
| AVR-PikE_Geraniol_uff_E=109.74 | -4.3 | 0 | 0 |
| AVR-PikE_Cinnamaldehyde_uff_E=76.51 | -4.2 | 0 | 0 |
| AVR-PikE_Trigonelline_uff_E=412.52 | -4.2 | 0 | 0 |
| AVR-PikE_Ajoene_uff_E=148.82 | -3.3 | 0 | 0 |
| AVR-PikE_Allicin_uff_E=111.53 | -3.1 | 0 | 0 |

| **Ligand** | **Binding Affinity** | **RMSD/UB** | **RMSD/LB** |
| --- | --- | --- | --- |
| AVR-PikF_Hecogenin_uff_E=836.11 | -6.5 | 0 | 0 |
| AVR-PikF_Rutin_uff_E=751.29 | -6.5 | 0 | 0 |
| AVR-PikF_Hesperedin_uff_E=589.61 | -6.4 | 0 | 0 |
| AVR-PikF_Betulinicacid_uff_E=790.90 | -6.3 | 0 | 0 |
| AVR-PikF_Luteolin_uff_E=241.99 | -6.2 | 0 | 0 |
| AVR-PikF_Quercetin_uff_E=379.98 | -6.1 | 0 | 0 |
| AVR-PikF_Berberine_uff_E=579.71 | -6 | 0 | 0 |
| AVR-PikF_CucurbitacinE_uff_E=864.84 | -5.9 | 0 | 0 |
| AVR-PikF_Rosmarinic_acid_uff_E=293.67 | -5.9 | 0 | 0 |
| AVR-PikF_Kaempferol_uff_E=362.50 | -5.8 | 0 | 0 |
| AVR-PikF_Naringenin_uff_E=195.82 | -5.8 | 0 | 0 |
| AVR-PikF_Catechin_uff_E=205.12 | -5.6 | 0 | 0 |
| AVR-PikF_Emodin_uff_E=196.49 | -5.6 | 0 | 0 |
| AVR-PikF_Ellagic_Acid_uff_E=215.94 | -5.5 | 0 | 0 |
| Strobuilirin | -5.4 |  |  |
| AVR-PikF_Resveratrol_uff_E=172.23 | -5.4 | 0 | 0 |
| AVR-PikF_Rotenone_uff_E=627.57 | -5.3 | 0 | 0 |
| AVR-PikF_Curcumin_uff_E=1321.66 | -5.1 | 0 | 0 |
| AVR-PikF_Turmerone_uff_E=144.16 | -4.9 | 0 | 0 |
| AVR-PikF_Matrine_uff_E=243.55 | -4.8 | 0 | 0 |
| AVR-PikF_Caffeicacid_uff_E=98.70 | -4.5 | 0 | 0 |
| AVR-PikF_Carvacrol_uff_E=78.47 | -4.5 | 0 | 0 |
| AVR-PikF_Ferulic_acid_uff_E=470.76 | -4.5 | 0 | 0 |
| AVR-PikF_Umbelliferone_uff_E=96.98 | -4.4 | 0 | 0 |
| AVR-PikF_Geraniol_uff_E=109.74 | -4.3 | 0 | 0 |
| AVR-PikF_Thymol_uff_E=95.99 | -4.2 | 0 | 0 |
| AVR-PikF_Thymoquinone_uff_E=62.47 | -4.2 | 0 | 0 |
| AVR-PikF_Gallicacid_uff_E=77.81 | -4.1 | 0 | 0 |
| AVR-PikF_D-Limonene_uff_E=101.93 | -4 | 0 | 0 |
| AVR-PikF_Eucalyptol_uff_E=344.57 | -4 | 0 | 0 |
| AVR-PikF_Paeonol_uff_E=127.65 | -4 | 0 | 0 |
| AVR-PikF_Cinnamaldehyde_uff_E=76.51 | -3.9 | 0 | 0 |
| AVR-PikF_Camphor_uff_E=400.52 | -3.8 | 0 | 0 |
| AVR-PikF_Trigonelline_uff_E=412.52 | -3.6 | 0 | 0 |
| AVR-PikF_Ajoene_uff_E=148.82 | -3.2 | 0 | 0 |
| AVR-PikF_Allicin_uff_E=111.53 | -2.6 | 0 | 0 |

| **Ligand** | **Binding Affinity** | **RMSD/UB** | **RMSD/LB** |
| --- | --- | --- | --- |
| AvrPiz-t_CucurbitacinE_uff_E=864.84 | -6.7 | 0 | 0 |
| AvrPiz-t_Hesperedin_uff_E=589.61 | -6.8 | 0 | 0 |
| AvrPiz-t_Hecogenin_uff_E=836.11 | -6.7 | 0 | 0 |
| strobulirin | -6.5 | 0 | 0 |
| AvrPiz-t_Betulinicacid_uff_E=790.90 | -6.5 | 0 | 0 |
| AvrPiz-t_Rutin_uff_E=751.29 | -6.4 | 0 | 0 |
| AvrPiz-t_Luteolin_uff_E=241.99 | -6.2 | 0 | 0 |
| AvrPiz-t_Berberine_uff_E=579.71 | -6.1 | 0 | 0 |
| AvrPiz-t_Naringenin_uff_E=195.82 | -6.1 | 0 | 0 |
| AvrPiz-t_Rotenone_uff_E=627.57 | -6.1 | 0 | 0 |
| AvrPiz-t_Quercetin_uff_E=379.98 | -6 | 0 | 0 |
| AvrPiz-t_Catechin_uff_E=205.12 | -5.9 | 0 | 0 |
| AvrPiz-t_Ellagic_Acid_uff_E=215.94 | -5.9 | 0 | 0 |
| AvrPiz-t_Kaempferol_uff_E=362.50 | -5.9 | 0 | 0 |
| AvrPiz-t_Emodin_uff_E=196.49 | -5.6 | 0 | 0 |
| AvrPiz-t_Rosmarinic_acid_uff_E=293.67 | -5.4 | 0 | 0 |
| AvrPiz-t_Turmerone_uff_E=144.16 | -5.4 | 0 | 0 |
| AvrPiz-t_Resveratrol_uff_E=172.23 | -5.3 | 0 | 0 |
| AvrPiz-t_Curcumin_uff_E=1321.66 | -5.2 | 0 | 0 |
| AvrPiz-t_Matrine_uff_E=243.55 | -5 | 0 | 0 |
| AvrPiz-t_Umbelliferone_uff_E=96.98 | -4.8 | 0 | 0 |
| AvrPiz-t_Caffeicacid_uff_E=98.70 | -4.7 | 0 | 0 |
| AvrPiz-t_Carvacrol_uff_E=78.47 | -4.7 | 0 | 0 |
| AvrPiz-t_Ferulic_acid_uff_E=470.76 | -4.7 | 0 | 0 |
| AvrPiz-t_Thymoquinone_uff_E=62.47 | -4.7 | 0 | 0 |
| AvrPiz-t_Thymol_uff_E=95.99 | -4.5 | 0 | 0 |
| AvrPiz-t_Gallicacid_uff_E=77.81 | -4.4 | 0 | 0 |
| AvrPiz-t_Paeonol_uff_E=127.65 | -4.4 | 0 | 0 |
| AvrPiz-t_Cinnamaldehyde_uff_E=76.51 | -4.3 | 0 | 0 |
| AvrPiz-t_D-Limonene_uff_E=101.93 | -4.2 | 0 | 0 |
| AvrPiz-t_Geraniol_uff_E=109.74 | -4.2 | 0 | 0 |
| AvrPiz-t_Camphor_uff_E=400.52 | -4.1 | 0 | 0 |
| AvrPiz-t_Eucalyptol_uff_E=344.57 | -4 | 0 | 0 |
| AvrPiz-t_Trigonelline_uff_E=412.52 | -3.8 | 0 | 0 |
| AvrPiz-t_Ajoene_uff_E=148.82 | -3.3 | 0 | 0 |
| AvrPiz-t_Allicin_uff_E=111.53 | -3.3 | 0 | 0 |

| **Ligand** | **Binding Affinity** | **RMSD/UB** | **RMSD/LB** |
| --- | --- | --- | --- |
| MAX47_Hesperedin_uff_E=589.61 | -8.7 | 0 | 0 |
| MAX47_CucurbitacinE_uff_E=864.84 | -8 | 0 | 0 |
| MAX47_Hecogenin_uff_E=836.11 | -7.9 | 0 | 0 |
| MAX47_Curcumin_uff_E=1321.66 | -7.3 | 0 | 0 |
| MAX47_Ellagic_Acid_uff_E=215.94 | -7.3 | 0 | 0 |
| MAX47_Berberine_uff_E=579.71 | -7.1 | 0 | 0 |
| MAX47_Rotenone_uff_E=627.57 | -7.1 | 0 | 0 |
| MAX47_Rutin_uff_E=751.29 | -7.1 | 0 | 0 |
| strobulirin | -6.9 | 0 | 0 |
| MAX47_Betulinicacid_uff_E=790.90 | -6.9 | 0 | 0 |
| MAX47_Emodin_uff_E=196.49 | -6.9 | 0 | 0 |
| MAX47_Luteolin_uff_E=241.99 | -6.9 | 0 | 0 |
| MAX47_Naringenin_uff_E=195.82 | -6.8 | 0 | 0 |
| MAX47_Catechin_uff_E=205.12 | -6.7 | 0 | 0 |
| MAX47_Kaempferol_uff_E=362.50 | -6.7 | 0 | 0 |
| MAX47_Quercetin_uff_E=379.98 | -6.7 | 0 | 0 |
| MAX47_Rosmarinic_acid_uff_E=293.67 | -6.4 | 0 | 0 |
| MAX47_Matrine_uff_E=243.55 | -6.1 | 0 | 0 |
| MAX47_Resveratrol_uff_E=172.23 | -6.1 | 0 | 0 |
| MAX47_Caffeicacid_uff_E=98.70 | -5.3 | 0 | 0 |
| MAX47_Gallicacid_uff_E=77.81 | -5.3 | 0 | 0 |
| MAX47_Turmerone_uff_E=144.16 | -5.2 | 0 | 0 |
| MAX47_Umbelliferone_uff_E=96.98 | -5.2 | 0 | 0 |
| MAX47_Ferulic_acid_uff_E=470.76 | -5.1 | 0 | 0 |
| MAX47_Thymoquinone_uff_E=62.47 | -5.1 | 0 | 0 |
| MAX47_Carvacrol_uff_E=78.47 | -5 | 0 | 0 |
| MAX47_Thymol_uff_E=95.99 | -5 | 0 | 0 |
| MAX47_Paeonol_uff_E=127.65 | -4.9 | 0 | 0 |
| MAX47_Camphor_uff_E=400.52 | -4.8 | 0 | 0 |
| MAX47_Cinnamaldehyde_uff_E=76.51 | -4.7 | 0 | 0 |
| MAX47_D-Limonene_uff_E=101.93 | -4.7 | 0 | 0 |
| MAX47_Eucalyptol_uff_E=344.57 | -4.5 | 0 | 0 |
| MAX47_Geraniol_uff_E=109.74 | -4.4 | 0 | 0 |
| MAX47_Trigonelline_uff_E=412.52 | -4.3 | 0 | 0 |
| MAX47_Ajoene_uff_E=148.82 | -3.9 | 0 | 0 |
| MAX47_Allicin_uff_E=111.53 | -3.5 | 0 | 0 |

| **Ligand** | **Binding Affinity** | **RMSD/UB** | **RMSD/LB** |
| --- | --- | --- | --- |
| MAX60__Hecogenin_uff_E=836.11 | -8.3 | 0 | 0 |
| MAX60__Rutin_uff_E=751.29 | -8.1 | 0 | 0 |
| MAX60__Hesperedin_uff_E=589.61 | -8 | 0 | 0 |
| MAX60__CucurbitacinE_uff_E=864.84 | -7.9 | 0 | 0 |
| MAX60__Rotenone_uff_E=627.57 | -7.5 | 0 | 0 |
| MAX60__Betulinicacid_uff_E=790.90 | -7.3 | 0 | 0 |
| MAX60__Rosmarinic_acid_uff_E=293.67 | -7.3 | 0 | 0 |
| Strobulirin | -6.9 | 0 | 0 |
| MAX60__Berberine_uff_E=579.71 | -7.2 | 0 | 0 |
| MAX60__Catechin_uff_E=205.12 | -7 | 0 | 0 |
| MAX60__Emodin_uff_E=196.49 | -6.9 | 0 | 0 |
| MAX60__Kaempferol_uff_E=362.50 | -6.9 | 0 | 0 |
| MAX60__Luteolin_uff_E=241.99 | -6.9 | 0 | 0 |
| MAX60__Naringenin_uff_E=195.82 | -6.9 | 0 | 0 |
| MAX60__Quercetin_uff_E=379.98 | -6.9 | 0 | 0 |
| MAX60__Matrine_uff_E=243.55 | -6.7 | 0 | 0 |
| MAX60__Ellagic_Acid_uff_E=215.94 | -6.5 | 0 | 0 |
| MAX60__Caffeicacid_uff_E=98.70 | -6.4 | 0 | 0 |
| MAX60__Umbelliferone_uff_E=96.98 | -6.4 | 0 | 0 |
| MAX60__Resveratrol_uff_E=172.23 | -6.3 | 0 | 0 |
| MAX60__Curcumin_uff_E=1321.66 | -6.2 | 0 | 0 |
| MAX60__Turmerone_uff_E=144.16 | -5.9 | 0 | 0 |
| MAX60__Gallicacid_uff_E=77.81 | -5.8 | 0 | 0 |
| MAX60__Carvacrol_uff_E=78.47 | -5.6 | 0 | 0 |
| MAX60__Ferulic_acid_uff_E=470.76 | -5.5 | 0 | 0 |
| MAX60__Paeonol_uff_E=127.65 | -5.3 | 0 | 0 |
| MAX60__Thymol_uff_E=95.99 | -5.3 | 0 | 0 |
| MAX60__D-Limonene_uff_E=101.93 | -5.2 | 0 | 0 |
| MAX60__Trigonelline_uff_E=412.52 | -5.2 | 0 | 0 |
| MAX60__Cinnamaldehyde_uff_E=76.51 | -5.1 | 0 | 0 |
| MAX60__Eucalyptol_uff_E=344.57 | -5.1 | 0 | 0 |
| MAX60__Thymoquinone_uff_E=62.47 | -5 | 0 | 0 |
| MAX60__Camphor_uff_E=400.52 | -4.9 | 0 | 0 |
| MAX60__Geraniol_uff_E=109.74 | -4.8 | 0 | 0 |
| MAX60__Ajoene_uff_E=148.82 | -3.9 | 0 | 0 |
| MAX60__Allicin_uff_E=111.53 | -3.5 | 0 | 0 |

| **Ligand** | **Binding Affinity** | **RMSD/UB** | **RMSD/LB** |
| --- | --- | --- | --- |
| MAX67__Hesperedin_uff_E=589.61 | -6.8 | 0 | 0 |
| MAX67__Rutin_uff_E=751.29 | -6.5 | 0 | 0 |
| MAX67__Hecogenin_uff_E=836.11 | -6.3 | 0 | 0 |
| MAX67__Rotenone_uff_E=627.57 | -6.1 | 0 | 0 |
| MAX67__CucurbitacinE_uff_E=864.84 | -5.8 | 0 | 0 |
| MAX67__Betulinicacid_uff_E=790.90 | -5.6 | 0 | 0 |
| MAX67__Curcumin_uff_E=1321.66 | -5.6 | 0 | 0 |
| MAX67__Rosmarinic_acid_uff_E=293.67 | -5.6 | 0 | 0 |
| MAX67__Ellagic_Acid_uff_E=215.94 | -5.5 | 0 | 0 |
| MAX67__Kaempferol_uff_E=362.50 | -5.5 | 0 | 0 |
| strobulirin | -5.4 |  |  |
| MAX67__Luteolin_uff_E=241.99 | -5.4 | 0 | 0 |
| MAX67__Naringenin_uff_E=195.82 | -5.4 | 0 | 0 |
| MAX67__Quercetin_uff_E=379.98 | -5.4 | 0 | 0 |
| MAX67__Berberine_uff_E=579.71 | -5.3 | 0 | 0 |
| MAX67__Matrine_uff_E=243.55 | -5.3 | 0 | 0 |
| MAX67__Emodin_uff_E=196.49 | -5.2 | 0 | 0 |
| MAX67__Catechin_uff_E=205.12 | -5.1 | 0 | 0 |
| MAX67__Resveratrol_uff_E=172.23 | -4.6 | 0 | 0 |
| MAX67__Turmerone_uff_E=144.16 | -4.6 | 0 | 0 |
| MAX67__Ferulic_acid_uff_E=470.76 | -4.5 | 0 | 0 |
| MAX67__Caffeicacid_uff_E=98.70 | -4.4 | 0 | 0 |
| MAX67__Umbelliferone_uff_E=96.98 | -4.4 | 0 | 0 |
| MAX67__Gallicacid_uff_E=77.81 | -4.3 | 0 | 0 |
| MAX67__Thymoquinone_uff_E=62.47 | -4.3 | 0 | 0 |
| MAX67__Carvacrol_uff_E=78.47 | -4.2 | 0 | 0 |
| MAX67__D-Limonene_uff_E=101.93 | -4.2 | 0 | 0 |
| MAX67__Camphor_uff_E=400.52 | -4.1 | 0 | 0 |
| MAX67__Geraniol_uff_E=109.74 | -4.1 | 0 | 0 |
| MAX67__Thymol_uff_E=95.99 | -4.1 | 0 | 0 |
| MAX67__Cinnamaldehyde_uff_E=76.51 | -4 | 0 | 0 |
| MAX67__Paeonol_uff_E=127.65 | -4 | 0 | 0 |
| MAX67__Eucalyptol_uff_E=344.57 | -3.9 | 0 | 0 |
| MAX67__Trigonelline_uff_E=412.52 | -3.7 | 0 | 0 |
| MAX67__Ajoene_uff_E=148.82 | -3.3 | 0 | 0 |
| MAX67__Allicin_uff_E=111.53 | -3.1 | 0 | 0 |
