# Supplementary material for: Exploring effector protein dynamics and natural fungicidal potential in rice blast pathogen Magnaporthe oryzae
Source: PLoS One. 2025 Jan 24;20(1):e0307352. doi: 10.1371/journal.pone.0307352 (PMC11761166; doi:10.1371/journal.pone.0307352)
Supplement: S1 Fig — All significant 2D interaction of ligand Hecogenin with protein A) APIKL2A, B) APIKL2F, C) AVRPIA, D) AVRPIB, E) AVRPII, F) AVRPIKA, G) AVRPIKC, H) AVRPIKD, I) AVRPIKE, J) AVRPIKF, K) AVRPIZT, L) MAX60, M) MAX47, N) MAX67. (DOCX) [file pone.0307352.s003.docx]

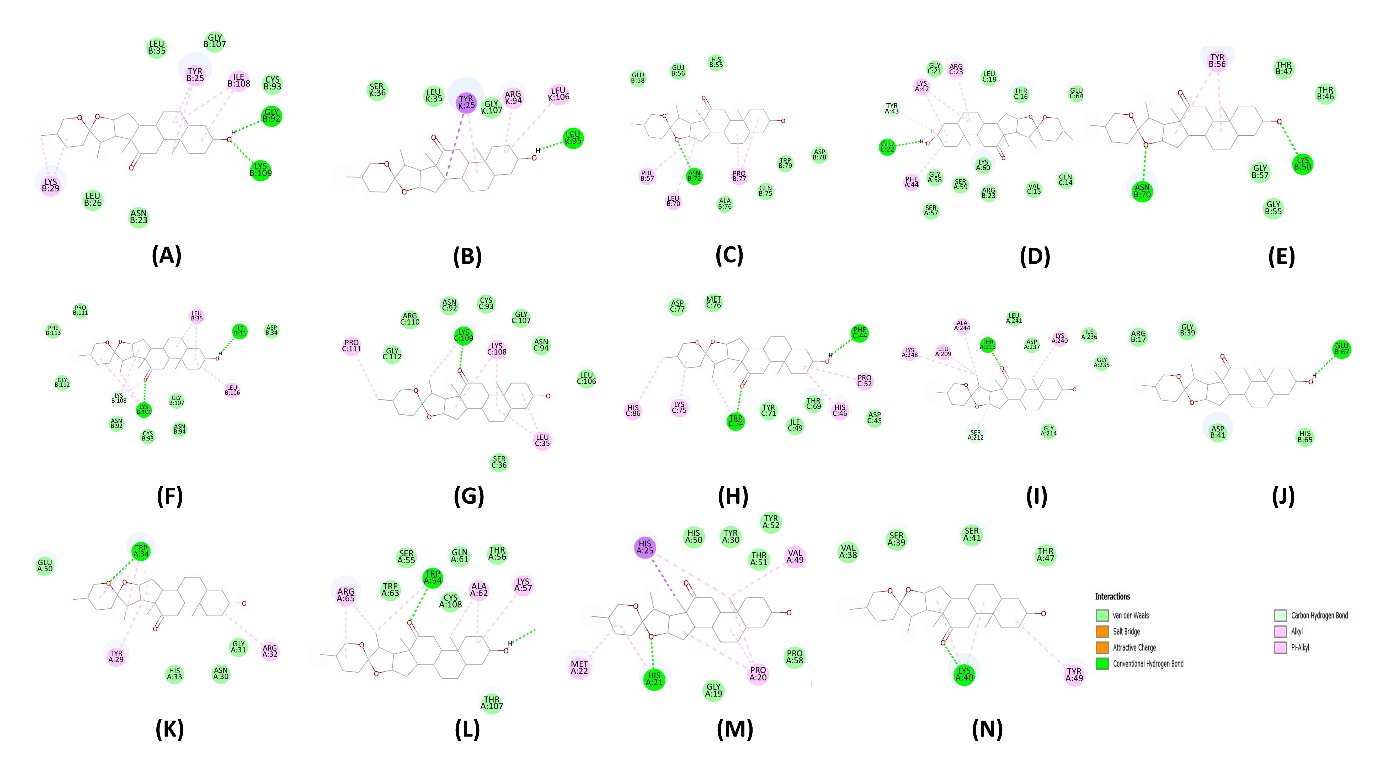


**Figure S1.** All significant 2D interaction of ligand Hecogenin with protein A)APIKL2A, B)APIKL2F, C)AVRPIA, D)AVRPIB, E)AVRPII, F)AVRPIKA, G)AVRPIKC, H)AVRPIKD, I)AVRPIKE, J)AVRPIKF, , K)AVRPIZT, L)MAX60, M)MAX47, N)MAX67
