# Supplementary material for: Exploring effector protein dynamics and natural fungicidal potential in rice blast pathogen Magnaporthe oryzae
Source: PLoS One. 2025 Jan 24;20(1):e0307352. doi: 10.1371/journal.pone.0307352 (PMC11761166; doi:10.1371/journal.pone.0307352)
Supplement: S3 Fig — A) ApikL-2a (HEC), B) Apikl-2a (STR), C) ApikL-2f (HEC), and D) ApikL-2f (STR). (DOCX) [file pone.0307352.s005.docx]

**
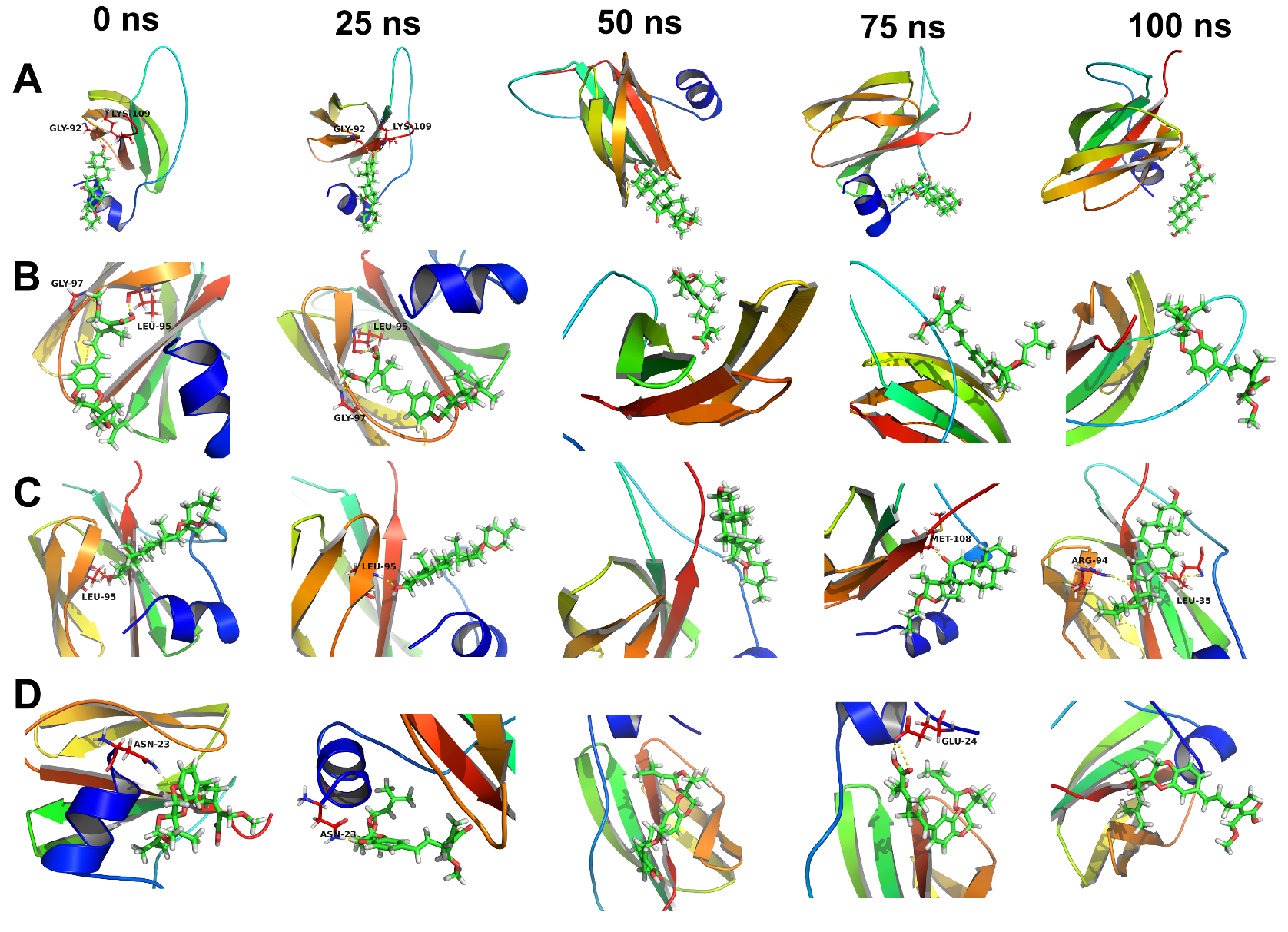
**

**Figure S3:** Hydrogen bond interactions in trajectories at different time intervals. A) ApikL-2a (HEC), B) Apikl-2a (STR), C) ApikL-2f (HEC), and D) ApikL-2f (STR)
