# Supplementary material for: Exploring effector protein dynamics and natural fungicidal potential in rice blast pathogen Magnaporthe oryzae
Source: PLoS One. 2025 Jan 24;20(1):e0307352. doi: 10.1371/journal.pone.0307352 (PMC11761166; doi:10.1371/journal.pone.0307352)
Supplement: S5 Fig — A) AVR-Pii (HEC), B) AVR-Pii (STR), C) AVR-PikA, and D) AVR-PikA (STR). (DOCX) [file pone.0307352.s007.docx]

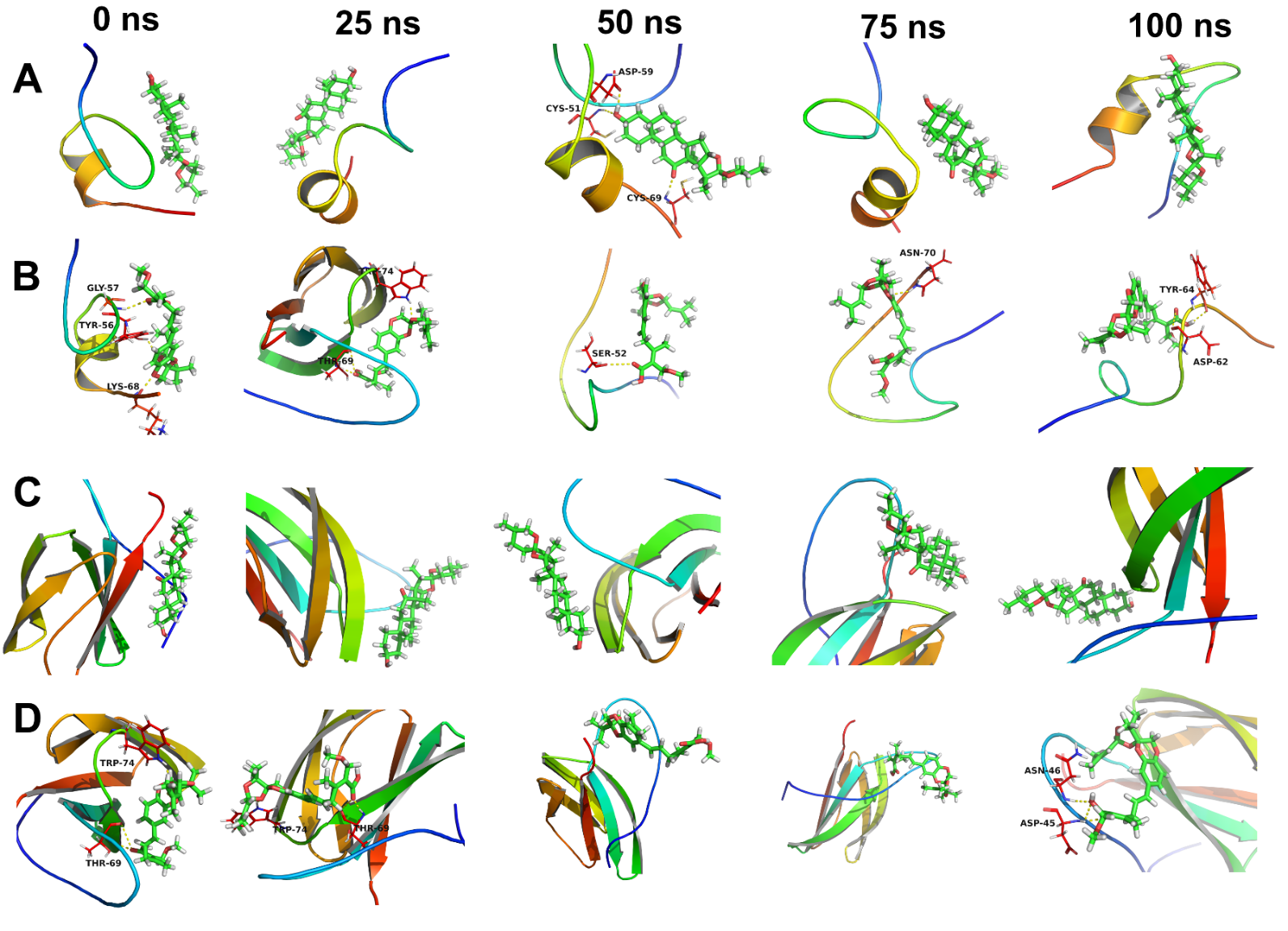


**Figure S5:** Hydrogen bond interactions in trajectories at different time intervals. A) AVR-Pii (HEC), B) AVR-Pii (STR), C) AVR-PikA, and D) AVR-PikA (STR)
