# Supplementary material for: Exploring effector protein dynamics and natural fungicidal potential in rice blast pathogen Magnaporthe oryzae
Source: PLoS One. 2025 Jan 24;20(1):e0307352. doi: 10.1371/journal.pone.0307352 (PMC11761166; doi:10.1371/journal.pone.0307352)
Supplement: S9 Fig — A) MAX60 (HEC), B) MAX60 (STR), C) MAX67 (HEC), and D) MAX67 (STR). (DOCX) [file pone.0307352.s011.docx]

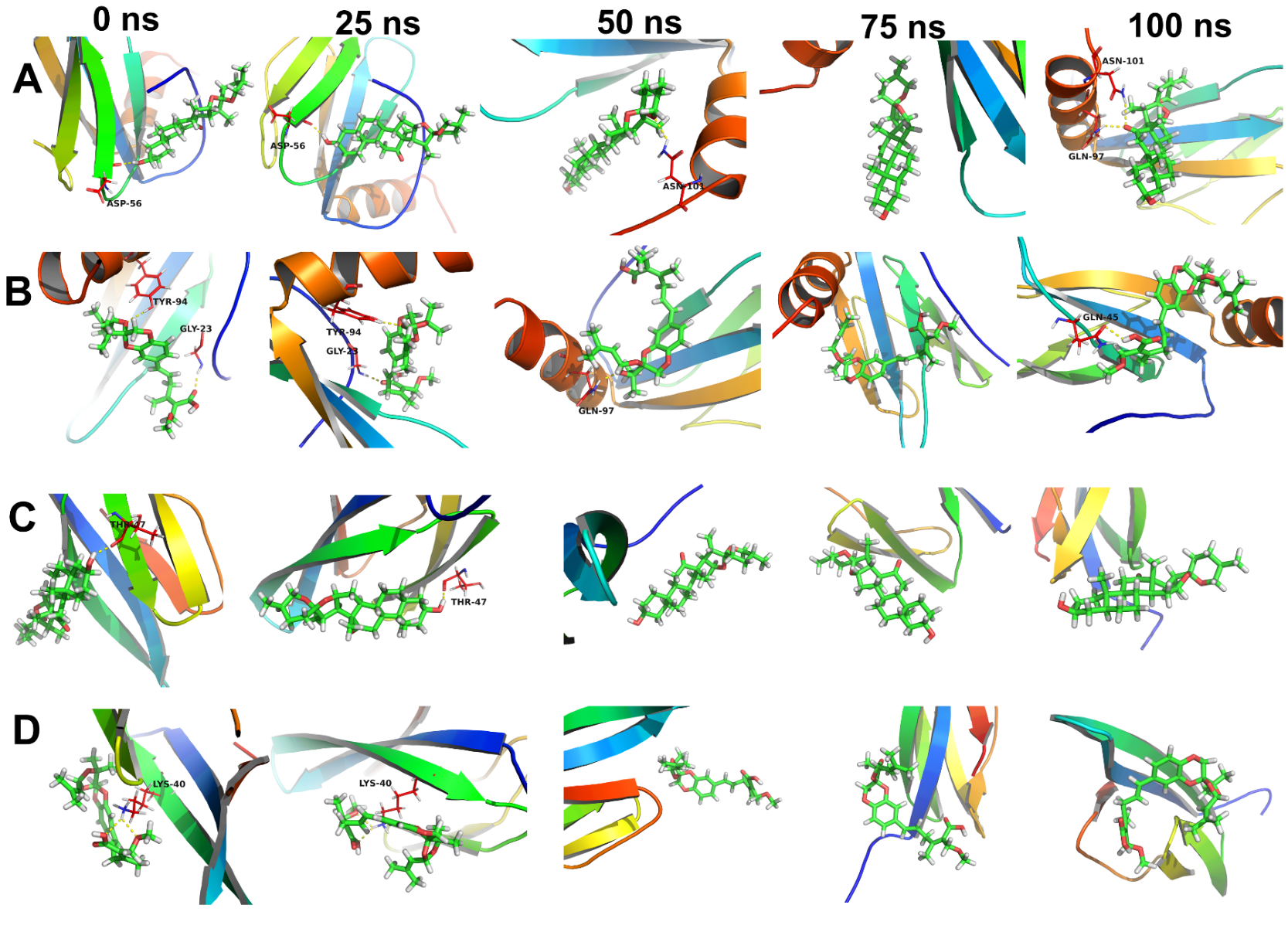


**Figure S9:** Hydrogen bond interactions in trajectories at different time intervals. A) MAX60 (HEC), B) MAX60 (STR), C) MAX67 (HEC), and D) MAX67 (STR)
