# Supplementary material for: Exploring effector protein dynamics and natural fungicidal potential in rice blast pathogen Magnaporthe oryzae
Source: PLoS One. 2025 Jan 24;20(1):e0307352. doi: 10.1371/journal.pone.0307352 (PMC11761166; doi:10.1371/journal.pone.0307352)
Supplement: Attachment — Submitted filename: Responses to the Reviewers.docx [file pone.0307352.s012.docx]

Date: 12.11. 2024

To

Editor-in-Chief

**PLOS ONE**

**Subject:** Submission of the revised manuscript

Dear Sir,

We have carried out essential revisions of our manuscript entitled “**Exploring Effector Protein Dynamics and Natural Fungicidal Potential in Rice Blast Pathogen *Magnaporthe oryzae***” suggested by the reviewer and would like to submit the 1^st^-revised version. We believe these comments/suggestions have significantly improved our manuscript. Please find enclosed the rebuttal letter where we have addressed all questions and comments to the reviewer, which are reflected in the tracked changes within the manuscript. Please be noted that all authors consented and approved the revised version of the manuscript. We believe the updated version will be worthy of publishing in your journal.

Thanking you in advance for considering our work, I remain,

Sincerely,

Abu Tayab Moin

Department of Genetic Engineering and Biotechnology,

Faculty of Biological Sciences, University of Chittagong, Chattogram, Bangladesh

Email: [tayabmoin786@gmail.com](mailto:tayabmoin786@gmail.com)

(Corresponding author on behalf of all authors)

**Author Responses to the Reviewers’ Comments**

**Reviewer #1:**

**Reviewer’s comment:** The authors focused on 35 plant-derived metabolites known for their antifungal properties, evaluating their potential as fungicidal agents against M. oryzae. Molecular docking analyses identified Hecogenin and Cucurbitacin E as highly effective binders to MAX40 and APIKL2A proteins, respectively, which are pivotal for fungal virulence and immune evasion. Molecular dynamics simulations further validated strong and stable interactions, affirming the therapeutic potential of these compounds. Additional assessments including Lipinski's rule of five criteria and toxicity predictions indicated their suitability for agricultural use. These findings underscore the promise of Hecogenin and Cucurbitacin E as lead candidates in developing novel fungicidal strategies against rice blast, offering prospects for enhanced crop protection and agricultural sustainability.

Comments:

1) The study seems to be technically well done.

2) However, the motivation is unclear; please explain why the study was done: If I know from the start that the 35 plant-derived metabolites are known for their antifungal properties, I do not need to do docking or MD to recognize this.

**The author(s) response:** Thank you very much for highlighting this point, as it provides an opportunity to clarify the primary motivation and unique objectives behind our study. We indeed agree that while the antifungal properties of these plant-derived metabolites have been documented in various literature sources, the specific molecular targets and mechanisms of action underlying their antifungal effects against *Magnaporthe oryzae* have not been fully elucidated. Our study aimed to address this gap by identifying specific target proteins in *M. oryzae* that may be effectively inhibited by these compounds, thereby providing a deeper understanding of the molecular mechanisms that confer antifungal activity.

To elaborate, molecular docking and molecular dynamics (MD) simulations offer several critical insights beyond simply confirming antifungal properties. Docking studies allow us to predict the preferred binding modes of small molecules within the binding pockets of specific proteins, thereby providing a molecular-level view of ligand-receptor interactions. Such insights are essential for understanding how these compounds interact with proteins that play key roles in *M. oryzae*'s pathogenicity and immune evasion, such as MAX40 and APIKL2A, which have not been extensively explored in previous antifungal research.

Our molecular docking analysis focused on screening these 35 compounds to evaluate their binding affinities with effector proteins in *M. oryzae*. This step was crucial for prioritizing compounds with the strongest interactions, such as Hecogenin and Cucurbitacin E. These two compounds were then subjected to more detailed MD simulations to assess the stability and dynamics of their binding interactions under conditions that more closely mimic biological environments. MD simulations further allowed us to validate the binding affinity predictions from docking by evaluating the stability, structural integrity, and conformational flexibility of the protein-ligand complexes over time.

**Additional Motivation for *In Silico* Approach:**

The application of in silico methods, such as molecular docking and MD simulations, offers distinct advantages when studying potential antifungal agents. These methods:

1. **Save Time and Resources:** They allow us to narrow down a large library of compounds quickly, identifying the most promising candidates without the time and cost associated with extensive in vivo or in vitro screening.
2. **Provide Mechanistic Insights:** By modeling the interactions at the atomic level, in silico techniques enable a detailed understanding of how these compounds interact with fungal proteins, which can guide the optimization of these compounds for fungicidal efficacy.
3. **Aid Targeted Compound Design:** Our findings offer foundational insights that can inform the design of derivatives or modifications to enhance the efficacy and specificity of these compounds.

In summary, while we started with a list of compounds known for their general antifungal properties, our goal was to identify specific molecular targets within *M. oryzae* and to elucidate how these compounds may exert their antifungal effects at a mechanistic level. We believe that this targeted approach can lead to the development of more effective and potentially specific antifungal agents for managing rice blast disease.

We appreciate your thoughtful suggestion, as it has enabled us to provide a clearer explanation of the motivation behind our research. We will incorporate this clarification into the manuscript to ensure that the motivation for our work is clearly conveyed to the readers.

Thank you once again for your valuable input and consideration.

**Reviewer’s comment:** Similarly, the ranking of the binding makes not much sense if not compared to actual experimental data on binding properties as measured. So either provide this by own experiments, but at least from literature.

**The author(s) response:** We appreciate your valuable feedback on the importance of validating the ranked binding affinities with experimental data for better accuracy and interpretation. We understand that without experimental corroboration, binding affinity predictions from computational studies alone may have limited real-world relevance. Therefore, we have taken the following actions to address this concern:

1. **Ranking and Docking Validation**:

The binding affinity rankings of the metabolites were generated using molecular docking in PyRx software, and the validity of the docking results was assessed through reference literature that supports the effectiveness of PyRx docking predictions. In our manuscript, we have now emphasized the validation procedures used for PyRx, including benchmark studies that demonstrate its reliability in ranking binding affinities of small molecules with protein targets. We have cited relevant literature to strengthen this point in the Methods section.

2. **Literature-Based Comparison**:

We have added a comparison between our computational findings and available experimental data from prior studies in the Discussion section. Specifically, we reference studies that report the experimental binding affinities of similar ligands with related target proteins. This provides a context for our docking results, helping to substantiate our rankings and their relevance to the potential fungicidal activity of the metabolites tested. Although direct experimental data on our specific metabolites and targets are limited, these comparisons lend support to our binding affinity predictions and highlight the potential efficacy of the ranked compounds.

3. **Future Experimental Plans**:

We recognize the importance of experimental validation to confirm computational results and intend to conduct in vitro binding affinity measurements and activity assays on promising candidates as part of our future research. This will provide additional insights into the binding mechanisms and efficacy of the selected compounds against rice blast fungus. We have mentioned these plans in the manuscript as a note in the Future Directions section, outlining our commitment to further experimental validation.

Thank you once again for your insightful suggestion, which has helped enhance the rigor and interpretability of our results.

**Reviewer’s comment:** The ranking by one docking method is usually not reliable, could you not use three independent methods and then tell the reader from the comparison the top binders.

**The author(s) response:** Thank you very much for your insightful suggestion regarding the use of multiple docking methods to improve ranking reliability. We fully appreciate that employing multiple docking approaches can provide a more comprehensive view, as different docking tools apply unique scoring functions and algorithms to evaluate binding affinities.

In this study, we chose AutoDock Vina as it combines empirical and knowledge-based components, using steric, hydrogen bonding, electrostatic, desolvation energies, and conformational entropy terms in its scoring function. This combination has proven effective for accurately predicting binding free energy in similar studies.

However, we acknowledge that cross-validation with multiple docking programs could offer a more robust ranking. Each tool's scoring function and parameter weighting differ, which might lead to variability in binding affinity rankings across methods. Thus, a compound ranked highly in one method may perform differently in another due to differences in algorithmic interpretation, which can sometimes complicate the consistency and interpretation of results.

Given the current scope and resources available for this project, we prioritized AutoDock Vina due to its reliability and its compatibility with our workflow. Implementing multiple docking programs would indeed add an extra layer of rigor; however, it may require additional computational resources and validation efforts that are beyond the scope of this study.

We respectfully hope the esteemed reviewer understands our rationale for focusing on AutoDock Vina for this study. We sincerely appreciate the suggestion and will consider incorporating multiple docking methods in future studies to further enhance our findings.

**Reviewer’s comment:** Here an interesting result is apparent: "Hecogenin and Cucurbitacin E

consistently exhibited the highest binding affinity across all 14 effector proteins. Specifically,

Hecogenin demonstrated the strongest binding energy with APIKL2A protein (-8.8 kJ/mol),

while Cucurbitacin E showed the highest binding energy with MAX40 protein (-8.0 kJ/mol).

Both compounds outperformed the reference metabolite, Strobilan, in binding affinity.

SwissADME server analysis confirmed their favorable phytochemical properties, suggesting

their potential as effective fungicides."

So again: what do the experiments say, is this known already and are both used as best drugs? If so, why was the study necessary?

**The author(s) response:** Thank you very much for this insightful comment. We appreciate the opportunity to clarify the novelty and significance of our findings.

Hecogenin and Cucurbitacin E are indeed known for their general antifungal properties, which have been reported in various studies. However, to the best of our knowledge, no prior research has specifically evaluated or applied these compounds as potential fungicidal agents targeting *Magnaporthe oryzae*, the causative agent of rice blast disease. Given the economic and agricultural significance of rice blast, it is essential to explore new therapeutic avenues targeting *M. oryzae* specifically.

In our study, we aimed to systematically assess whether Hecogenin and Cucurbitacin E could effectively inhibit the specific effector proteins and virulence factors critical for *M. oryzae*'s pathogenicity. By targeting the key effectors APIKL2A and MAX40, we were able to reveal that these compounds demonstrated binding affinities significantly higher than those of Strobilurin, a known fungicidal reference compound. This finding highlights the unique potential of Hecogenin and Cucurbitacin E in disrupting the molecular mechanisms that enable *M. oryzae* infection.

Additionally, our comprehensive analysis includes molecular dynamics simulations and ADME/T (absorption, distribution, metabolism, excretion, and toxicity) predictions, which offer critical insights into the stability, efficacy, and safety of Hecogenin and Cucurbitacin E for use in agricultural applications. Our study represents the first report to screen and identify these compounds as lead candidates with specific activity against *M. oryzae*, potentially paving the way for their development as fungicidal agents targeting rice blast disease.

In summary, while the antifungal properties of Hecogenin and Cucurbitacin E have been generally recognized, our study establishes their specific application and binding efficacy against rice blast effectors, which has not been previously reported. We believe this contribution provides a meaningful advancement in the search for novel fungicidal agents against rice blast disease and hope the reviewer finds this explanation satisfactory.

Thank you once again for your valuable feedback.

**Reviewer’s comment:** May be you can explain why these two proteins bind well to all 14 effector proteins. Obviously, these have a related structure. Do all known effector proteins have a similar structure? If so, then resistance development for the pathogen is easy possible. Hence, can you point out an effector protein with different structure, which then could be targeted by second drug and prevent resistance development? Like this, the motivation for the study would also be more clear.

**The author(s) response:** Thank you for your insightful and constructive comment. We appreciate your thoughtful suggestion regarding the structural similarity of effector proteins and the potential for resistance development in the pathogen. We would like to address your concerns in detail:

1. **Binding to All 14 Effector Proteins**: Indeed, the effector proteins in M. oryzae exhibit homologous sequences and share structural similarities, which is why the compounds we identified in this study, such as Hecogenin and Cucurbitacin E, bind effectively to multiple effector proteins. This homology facilitates similar binding modes across the 14 effector proteins studied, which might give the impression that resistance could arise quickly if only one effector protein were targeted. However, this characteristic also underscores the advantage of targeting multiple effector proteins, as this approach reduces the likelihood of resistance development based on a single mutation in one effector protein.
2. **Structural Similarity and Resistance Development**: While the effector proteins share structural homology, it is important to note that their precise conformations and specific residues involved in ligand binding can vary, albeit slightly, across different effectors. This variance allows us to design metabolites that can bind with high affinity to multiple effectors, thus minimizing the risk of resistance. By targeting all 14 effector proteins, we aim to achieve broad-spectrum inhibition, making it more difficult for the pathogen to develop resistance.
3. **Structural Diversity in the Study**: As pointed out in the manuscript, there is structural diversity in both the metabolites used (Hecogenin and Cucurbitacin E) and the effector proteins. For instance, while the effector proteins share some common structural elements, they each possess unique features that were taken into account during the molecular docking and dynamics simulations. This structural diversity in both targets and ligands is one of the key reasons why these compounds exhibit varying degrees of binding affinity and stability across different effector proteins. We believe this variability contributes to the robustness of our approach in preventing the development of resistance.
4. **Alternative Strategy for Resistance Management**: Regarding your suggestion of targeting a structurally distinct effector protein with a second drug to prevent resistance development, we believe this is a valid approach. However, given the homology among the effector proteins and their role in the infection process, we have opted to target multiple effectors in this study. Nevertheless, we agree that identifying a structurally distinct effector protein, which could serve as a secondary target, is an intriguing strategy for further investigation. Such an approach could be explored in future studies to enhance our resistance management strategy.
5. **Study Motivation**: The motivation for this study stems from the need to develop a broad-spectrum fungicide capable of targeting multiple effector proteins, thus reducing the risk of resistance development. Our goal is to propose two promising natural metabolites, Hecogenin and Cucurbitacin E, as potential lead compounds for fungicide development. These compounds show promise due to their binding affinities with multiple effector proteins and their favorable physicochemical properties, which could contribute to their efficacy and safety as fungicidal agents. By targeting a range of effector proteins with structurally diverse compounds, we aim to develop more effective and sustainable strategies for controlling rice blast disease.

We hope this explanation provides more clarity on the rationale behind targeting multiple effector proteins and the potential for resistance management. We appreciate the reviewer's suggestions and will consider them in future studies to explore even more effective strategies for combating rice blast disease.

Thank you once again for your valuable feedback.

**Reviewer #2:**

**Reviewer’s comment:** This study identified Hecogenin and Cucurbitacin E as potent antifungal agents against M. oryzae through extensive bioinformatics analysis, including molecular docking and dynamics simulations. been no chemical or biological experimental verification, so the guidance for readers is not very significant. Therefore, I do not recommend publication and suggest adding verification experiments.

**The author(s) response:** Thank you for your thoughtful feedback. We understand the concern regarding the absence of experimental validation in our study. However, we would like to highlight that computational techniques such as molecular docking and molecular dynamics (MD) simulations are well-established and widely used in the early stages of drug discovery and development, particularly when evaluating potential inhibitors for diseases like rice blast.

Computational methods allow us to efficiently screen large numbers of compounds in a relatively short time, predicting their binding affinity and stability with the target proteins. This in silico approach serves as an essential first step in drug discovery by identifying promising candidates that can later be tested experimentally. In the context of our study, we identified Hecogenin and Cucurbitacin E as potential inhibitors of *Magnaporthe oryzae* based on their favorable binding profiles with effector proteins involved in rice blast pathogenesis.

While experimental validation is, of course, critical, computational predictions can guide the selection of compounds for experimental testing, potentially saving both time and resources by focusing efforts on the most promising candidates. In fact, numerous studies have successfully used molecular docking and MD simulations to identify lead compounds that were later confirmed to have biological activity in experimental assays. These computational approaches help reduce the number of compounds that need to be tested experimentally, thus improving the efficiency of the discovery process.

Moreover, the use of ADMET predictions in our study provides an additional layer of validation for the compounds identified, assessing their pharmacokinetic properties and safety profiles before moving to laboratory testing. This information is crucial for understanding how these compounds might behave in a biological system and their potential for further development as antifungal agents.

In summary, while experimental validation is an essential next step, we believe that the computational analysis presented in this study is both valid and useful as an early screening tool. The results provide a strong rationale for further experimental studies and offer valuable insights into potential candidates for the development of novel fungicides against rice blast.

We appreciate the reviewer’s feedback and hope that this explanation clarifies the role and value of our computational approach in identifying potential fungicide candidates.

**Reviewer #3:**

**Reviewer’s comment:** The manuscript demonstrates an impressive and proficient use of Computer-Aided Drug Design (CADD) in the development of novel pesticides, showcasing the potential of this approach in agricultural applications, there still are some revisions require clarification

1. The background information on Hecogenin, Cucurbitacin E is insufficient. Could you provide more context or expand on its origins, biological properties, and applications in research or pharmaceuticals? This will help to clarify its significance and relevance in the current context.

**The author(s) response:**

Thank you for your valuable feedback. We appreciate your suggestion to expand the background information on Hecogenin and Cucurbitacin E. We agree that providing a more comprehensive context on these compounds will help clarify their relevance in the current study. Below is the expanded information, which we plan to include in the introduction and discussion sections:

**Hecogenin** is a steroidal saponin derived primarily from the *Agave* species, including *Agave sisalana* and *Agave aurea*. It has been widely studied for its significant biological activities, including anticancer, antifungal, and hypotensive properties. Hecogenin acts as a selective inhibitor of human UDP-glucuronosyltransferases (UGTs), a class of enzymes involved in the detoxification of various drugs and toxins. This property makes Hecogenin a valuable candidate for enhancing drug metabolism and overcoming drug resistance, a critical issue in pharmacology. Furthermore, its antifungal properties suggest potential applications in the treatment of fungal infections, which aligns with its role in this study as a promising fungicidal agent against *Magnaporthe oryzae*, the causal agent of rice blast disease. The compound’s ability to target key enzymatic pathways and its diverse biological effects make it a noteworthy bioactive compound in drug design and agricultural research [1-3].

**Cucurbitacin E**, on the other hand, is a tetracyclic triterpenoid compound found predominantly in cucurbitaceous plants, such as pumpkins, gourds, and cucumbers. It is known for a wide range of biological activities, including antitumor, hepatoprotective, anti-inflammatory, and antioxidant effects. Cucurbitacin E inhibits the growth of various cancer cells and has shown protective effects on the liver, making it a promising candidate for therapeutic applications. Additionally, its anti-inflammatory properties are attributed to the suppression of pathways such as NF-κB, which plays a central role in regulating immune responses and inflammation. Due to its multiple biological effects, Cucurbitacin E has garnered interest in the fields of cancer research, liver disease, and inflammation. In the context of this study, Cucurbitacin E’s ability to inhibit key effector proteins involved in the infection process of *M. oryzae* further underscores its potential as a novel antifungal agent [4-6].

Both Hecogenin and Cucurbitacin E are derived from plant species with natural defense mechanisms, and their promising biological properties provide a strong foundation for their use in the development of new therapeutic agents. The insights gained from studying these compounds in the context of fungal infections, particularly rice blast disease, suggest that they may offer a new approach to fungicide development or serve as lead compounds for further optimization in crop protection strategies.

We hope that this additional context will help clarify the significance and relevance of Hecogenin and Cucurbitacin E in the current research. Thank you again for your constructive comments.

**References**

1. da Hora Borges, M. A., Passos, F. R. S., Quintans, J. D. S. S., & Azeredo, F. J. (2023). Hecogenin and its derivates: A pharmacology review. *Biomedicine & Pharmacotherapy*, *159*, 114251.

2. Ingawale, D. K., Mandlik, S. K., & Patel, S. S. (2019). Anti-inflammatory potential of hecogenin on atopic dermatitis and airway hyper-responsiveness by regulation of pro-inflammatory cytokines. *Immunopharmacology and Immunotoxicology*, *41*(2), 327-336.

3. Cerqueira, G. S., e Silva, G. D. S., Vasconcelos, E. R., de Freitas, A. P. F., Moura, B. A., Macedo, D. S., ... & de Barros Viana, G. S. (2012). Effects of hecogenin and its possible mechanism of action on experimental models of gastric ulcer in mice. *European journal of pharmacology*, *683*(1-3), 260-269.

4. Chung, S. O., Kim, Y. J., & Park, S. U. (2015). An updated review of cucurbitacins and their biological and pharmacological activities. *EXCLI journal*, *14*, 562.

5. Ramezani, M., Hasani, M., Ramezani, F., & Abdolmaleki, M. K. (2020). Cucurbitacins: A focus on Cucurbitacin E as a natural product and their biological activities. *Pharmaceutical Sciences*, *27*(1), 1-13.

6. Zhang, L., Liang, H., & Xin, Y. (2020). Cucurbitacin E inhibits esophageal carcinoma cell proliferation, migration, and invasion by suppressing Rac1 expression through PI3K/AKT/mTOR pathway. *Anti-cancer drugs*, *31*(8), 847-855.

**Reviewer’s comment:** The results part mainly introduced hydrogen bonding between small molecules and polar amino acids in the protein, and while hydrogen bonds do indeed play a significant role, might there be other interactions, such as π-π stacking or hydrophobic forces, that contribute to the interaction between the two?

**The author(s) response:** Thank you for your insightful comment regarding the types of interactions that contribute to the binding affinity between the small molecules and proteins. We appreciate your suggestion to consider additional non-bonded interactions, such as π-π stacking and hydrophobic forces, in our analysis. We fully agree that hydrogen bonds are only one aspect of the binding interactions, and other forces such as hydrophobic interactions, π-π stacking, and electrostatic forces play significant roles in the stability of protein-ligand complexes. These interactions are critical for optimizing the binding affinity and specificity of ligands to their target proteins.

As you correctly noted, we have revised the manuscript to include a broader discussion in section 3.3 and in Table 1, highlighting not only hydrogen bonding but also hydrophobic interactions, π-π stacking, and additional polar interactions between the ligands and target proteins. These supplementary interactions, such as the hydrophobic forces between non-polar residues of the ligands and proteins, contribute substantially to the overall binding strength. In particular, π-π stacking interactions, especially in ligand-protein complexes with aromatic residues in the binding sites, provide further stability.

In response to your suggestion, we have also added Supplementary Figures to visually represent these additional interactions, providing a more comprehensive view of the binding mechanisms. Supplementary Figure S1 presents all significant 2D interactions of the ligand Hecogenin with the proteins, including A) APIKL2A, B) APIKL2F, C) AVRPIA, D) AVRPIB, E) AVRPII, F) AVRPIKA, G) AVRPIKC, H) AVRPIKD, I) AVRPIKE, J) AVRPIKF, K) AVRPIZT, L) MAX60, M) MAX47, and N) MAX67. Similarly, Supplementary Figure S2 displays the key 2D interactions of Cucurbitacin E with the same protein targets. These figures allow for a detailed visualization of both hydrogen bonding and other significant interactions, including π-π stacking and hydrophobic forces.

We believe these additions, including the updated Table 1 and Supplementary Figures S1 and S2, provide a clearer understanding of the binding interactions and strengthen the manuscript by offering a more complete analysis of the molecular interactions involved in the binding of Hecogenin and Cucurbitacin E with the effector proteins.

**Figure S1.** All significant 2D interaction of ligand Hecogenin with protein A)APIKL2A, B)APIKL2F, C)AVRPIA, D)AVRPIB, E)AVRPII, F)AVRPIKA, G)AVRPIKC, H)AVRPIKD, I)AVRPIKE, J)AVRPIKF, , K)AVRPIZT, L)MAX60, M)MAX47, N)MAX67

**Figure S2.** All significant 2D interaction of ligand Cucurbitacin E with protein A)APIKL2A, B)APIKL2F, C)AVRPIA, D)AVRPIB, E)AVRPII, F)AVRPIKA, G)AVRPIKC, H)AVRPIKD, I)AVRPIKE, J)AVRPIKF, , K)AVRPIZT, L)MAX60, M)MAX47, N)MAX67

Thank you once again for your thoughtful suggestion. We look forward to any further comments you may have.

**Reviewer’s comment:** There may not be direct studies or predictions specifically addressing the toxicity of Hecogenin and Cucurbitacin E on rice plants. It would be advisable to conduct detailed phytotoxicity assays to prepare for further development into pesticides.

**The author(s) response:** Thank you for your thoughtful comment regarding the potential phytotoxicity of Hecogenin and Cucurbitacin E on rice plants. We appreciate your attention to this important aspect of pesticide development.

As you rightly pointed out, while our study has extensively addressed the molecular docking, molecular dynamics simulations, and toxicity analysis for these compounds in terms of their potential fungicidal properties and safety profiles, direct studies on their phytotoxicity in rice plants have not been conducted yet. We fully recognize that assessing the phytotoxicity of candidate fungicides is a critical step before advancing to field applications.

In response to your valuable suggestion, we plan to extend this work to include detailed phytotoxicity assays as the next phase of our research. This will involve evaluating the effects of Hecogenin and Cucurbitacin E on rice plant growth, leaf chlorosis, and other physiological responses, which will be essential for confirming their safety profile and ensuring they do not adversely impact the crop itself. We also aim to determine the effective concentrations at which these compounds are toxic to the pathogen but do not harm the plant, as this balance is crucial for the development of an effective, crop-safe fungicide.

Furthermore, we are in the process of refining our experimental protocols for these phytotoxicity assays, and we will ensure that the assays are conducted under controlled environmental conditions to simulate realistic field scenarios. This additional layer of testing will be important in validating the safety of these compounds for potential agricultural use.

We value your input and agree that phytotoxicity data are necessary to fully assess the potential of Hecogenin and Cucurbitacin E as future pesticide candidates. We are committed to conducting these studies in due course and will include these findings in subsequent publications or revisions.

Once again, thank you for your constructive feedback, and we look forward to addressing this aspect of our research in future work.
