# Supplementary material for: Exploring effector protein dynamics and natural fungicidal potential in rice blast pathogen Magnaporthe oryzae
Source: PLoS One. 2025 Jan 24;20(1):e0307352. doi: 10.1371/journal.pone.0307352 (PMC11761166; doi:10.1371/journal.pone.0307352)
Supplement: Attachment — Submitted filename: Responses to the Editor.docx [file pone.0307352.s013.docx]

Date: 30.12. 2024

To

Editor-in-Chief

**PLOS ONE**

**Subject:** Submission of the revised manuscript

Dear Sir,

We have carried out essential revisions of our manuscript entitled “**Exploring Effector Protein Dynamics and Natural Fungicidal Potential in Rice Blast Pathogen *Magnaporthe oryzae***” suggested by the reviewer and would like to submit the 2^nd^-revised version. We believe these comments/suggestions have significantly improved our manuscript. Please find enclosed the rebuttal letter where we have addressed all questions and comments to the reviewer, which are reflected in the tracked changes within the manuscript. Please be noted that all authors consented and approved the revised version of the manuscript. We believe the updated version will be worthy of publishing in your journal.

Thanking you in advance for considering our work, I remain,

Sincerely,

Abu Tayab Moin

Department of Genetic Engineering and Biotechnology,

Faculty of Biological Sciences, University of Chittagong, Chattogram, Bangladesh

Email: [tayabmoin786@gmail.com](mailto:tayabmoin786@gmail.com)

(Corresponding author on behalf of all authors)

**Author Responses to the Editor’s Comment**

**Reviewer’s comment:** Please add a short paragraph about the limitations of the molecular docking approach (without any experimental confirmation). Then the manuscript could be accepted for publication.

**The author(s) response:** We sincerely thank you for their insightful suggestion. We agree that discussing the limitations of the molecular docking approach adds depth and context to our study, highlighting the importance of further experimental validation. In response, we have added the following paragraph to the manuscript as per your recommendation:

*"However, the molecular docking approach has inherent limitations. It relies on static structural models and scoring algorithms that may not accurately reflect the dynamic and complex nature of protein-ligand interactions in physiological conditions. Furthermore, the lack of experimental confirmation restricts the ability to evaluate the true binding affinity, bioavailability, and effectiveness of these compounds in real biological systems. While our findings provide a promising foundation, further in vitro and in vivo studies are essential to validate the efficacy and safety of these compounds for practical applications."*

We believe this addition addresses your concern by acknowledging the methodological limitations while emphasizing the potential of our findings as a basis for future experimental investigations. Thank you once again for your valuable input, which has enhanced the quality of our manuscript.
